# High Resolution Postmortem MRI Discovers Developing Structural Connectivity in the Human Ascending Arousal Network

**DOI:** 10.1002/hbm.70422

**Published:** 2025-11-29

**Authors:** Roxane Licandro, Mark Olchanyi, Luiz F. Ferraz da Silva, Andre van der Kouwe, Camilo Jaimes, Nathan X. Ngo, William Kelley, Rebecca Folkerth, Robin L. Haynes, Brian L. Edlow, Hannah C. Kinney, Lilla Zöllei

**Affiliations:** ^1^ Laboratories for Computational Neuroimaging Athinoula A. Martinos Center for Biomedical Imaging, Massachusetts General Hospital and Harvard Medical School Charlestown Massachusetts USA; ^2^ Computational Imaging Research ‐ Early Life Image Analysis Group, Department of Biomedical Imaging and Image‐Guided Therapy Medical University of Vienna Vienna Austria; ^3^ Center for Neurotechnology and Neurorecovery, Department of Neurology Massachusetts General Hospital and Harvard Medical School Boston Massachusetts USA; ^4^ Neuroscience Statistics Research Laboratory Massachusetts Institute of Technology Cambridge Massachusetts USA; ^5^ University of São Paulo Department of Pathology São Paulo Brazil; ^6^ Department of Radiology Massachusetts General Hospital Boston Massachusetts USA; ^7^ Brain Injury Research Center, Icahn School of Medicine at Mount Sinai New York New York USA; ^8^ Department of Pathology Boston Children's Hospital Boston Massachusetts USA

**Keywords:** ascending arousal network, developing structural connectivity, developing tractography, fetal and infant imaging, graph‐based analysis, postmortem brain imaging

## Abstract

Human arousal is essential to survival and mediated by the ascending arousal network (AAN) and its connections. It spans from the brainstem to the diencephalon, basal forebrain, and cerebral cortex. Despite advances in mapping the AAN in adults, it is unexplored in fetal and early infant life, especially with high‐resolution magnetic resonance imaging techniques. In this study, we conducted—for the first time—high‐resolution ex vivo diffusion MRI‐based analysis of the AAN in seven fetal, infant, and adult brains, incorporating probabilistic tractography and quantifying connectivity using graph theory. We observed that AAN structural connectivity becomes increasingly integrated during development, progressively reaching rostrally during the first postconceptional year. We quantitatively identified the dorsal raphe (DR) nucleus and ventral tegmental area (VTA) as AAN connectivity hubs already in the fetus persisting into adulthood. The DR appears to form a local hub of short‐range connectivities, while the VTA evolves as a long‐range global hub. The identified connectivity maps advance our understanding of AAN architecture changes due to normative human brain development, as well as disorders of arousal, such as coma and sudden infant death syndrome.

## Graphical abstract

We used high‐resolution ex vivo diffusion MRI and graph theory to analyze ascending arousal network development in fetal to adult brains, revealing increasing rostral integration postnatally and identifying the dorsal raphe nucleus and ventral tegmental area as persistent connectivity hubs from fetal stages through adulthood.

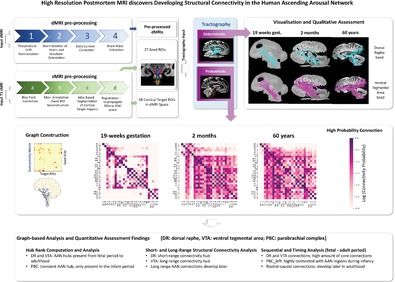

## 1 Introduction

Arousal is essential for human survival and evolutionary success. Human disorders of arousal affect all ages with a spectrum of state impairment ranging from excessive sleepiness to coma. Of special interest to public health are arousal disorders of early life, such as prenatal fetal suppression of active periods by maternal use of illicit drugs (Ross et al. 2015) and sudden infant death syndrome (SIDS). SIDS, in particular, is the leading cause of postneonatal infant mortality in the United States today, accounting for 0.33/1000 live births per year (Raven 2018a). SIDS is postulated to result from a central failure of arousal to life‐threatening homeostatic challenges during sleep, such as hypoxia or hypotension, that likely originate during fetal life and are expressed as sleep‐related sudden death in a critical postnatal period of brain development that is, the first postnatal year (Kinney and Haynes 2019). It has been suggested that the human fetus spends most of his/her time suspended in the womb's amniotic fluid in the unconscious state: asleep, unaware, and intermittently aroused. Two bulwarks of the conscious state have been proposed: awareness and arousal (Koch et al. 2016), each governed by different yet integrated neural networks (Edlow et al. 2024), with arousal defined as the responsiveness of both somatic and autonomic motor processes to external stimuli (Jones 2020). However, decades of investigation have revealed that the physiological characteristics of human wakefulness and sleep evolve as early as the second half of gestation and into the neonatal period and early infancy (Chang et al. 2022; Crossley et al. 2014; Fornito et al. 2015; Oldham and Fornito 2019). Significant and disparate changes in the organization of sleep and waking states occur in toddlers, children, adolescents, adults, and the elderly (Kinney et al. 1988). Fundamental questions arise: What constitutes arousal in early life? When do markers of what is considered “arousal” emerge in the fetus? Is there a critical period of arousal maturation in the first year of life, the period of SIDS risk? How does pathology disrupt components of arousal? The goal of this study is to establish a baseline regarding the development of neural network architectures and underlying neural correlates of arousal processes essential for answering these questions.

**FIGURE 1:**
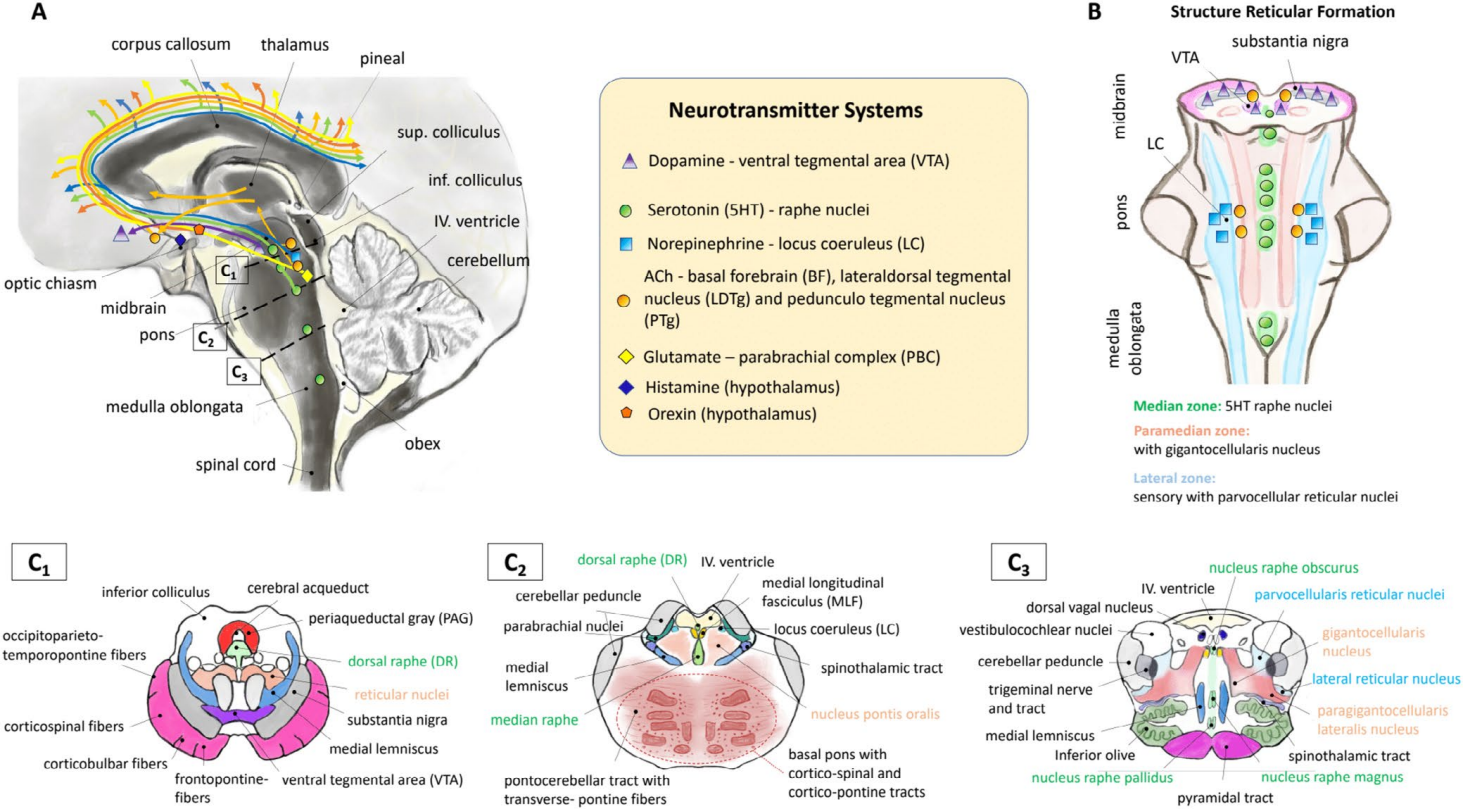
Schematic drawing of the ascending arousal network. (A) illustrates the neuromodulatory system for AcetylCholine (ACh), Dopamine (DA), Norepinephrine (NE) and Serotonin (5HT). Related anatomical projections from the brainstem, midbrain regions and cortical areas are also displayed. The broken lines visualize the cuts for the axial views visualized in subfigure C1—C3. In (B) the reticular formation is visualized with its three structurally defined zones: (1) the median zone with the raphe nuclei [dorsal raphe (see axial view C1), median raphe (see axial view C2) raphe pallidus, raphe obscurus, raphe magnus (see C3)], (2) the medial zone with the gigantocellular reticular nuclei (see axial view C3) and (3) the lateral zone with the parvocellular reticular nuclei (see axial view C3).

Arousal is mediated by the reticular formation with multiple ascending projections, for example, to the ponto‐mesencephalic tegmentum of the brainstem, hypothalamus, and basal forebrain (see Figure 1A) (Edlow et al. 2012; Parvizi and Damasio 2001). We designate this system as the “ascending arousal network” (AAN), which serves as the central focus of our study. Although, the neurons of the AAN are diffusely distributed in delimited regions (nuclei), these follow a structured arrangement particularly in the reticular formation (see Figure 1B). While it is predominantly serotonergic neurons, like the dorsal raphe (DR), median raphe (MnR) (see Figure 1_1_,C_2_), the nucleus raphe obscurus and nucleus raphe pallidus (see Figure 1C_3_), that are arranged along the *median zone* of the reticular formation, the *lateral zone* contains noradrenergic neurons in the locus coeruleus (see Figure 1C_2_). Also dopaminergic, cholinergic, glutamatergic, GABA‐ergic, histaminergic, and orexinergic neurons are part of the AAN (see overview of neurotransmitter systems and involved nuclei in Figure 1A). Awareness requires the integration of typically visual or auditory perceptual input and recall of memories of stored aspects of sensory input (Harper et al. 2002). Arousal is essential for awareness: without arousal, awareness, mediated largely by thalamocortical circuits, is not functional, and survival is compromised. Consciousness can, therefore, be considered a “progressive, stepwise, structural, and functional evolution of its multiple intricate components” (Padilla and Lagercrantz 2020), which are controversial and currently debated (Boly et al. 2017). Considering the neural correlates of consciousness in its own right is beyond the scope of this study. Nevertheless, our findings about the AAN structural development are highly relevant to understanding the underpinnings of human consciousness. In the adult human, integrative nodes and their vast interconnections have begun to be mapped (Jones 2020; Morris et al. 2008; Niederkofler et al. 2015; Tomasello 2019; Zhang et al. 2020). In contrast, little information about AAN connectivity is available in early human life that is, the critical period when consciousness emerges. Moreover, even less is known about the maturational sequences of AAN connectivity across the human lifespan. A major obstacle to acquiring such knowledge is the difficulty in dissecting the extraordinarily complex structural correlates of arousal with currently available anatomic tools, as interconnectivity between relevant structures is nearly impossible to visualize *in toto* with standard neuropathologic techniques at autopsy. Even promising tools, such as CLARITY (https://picower.mit.edu/innovations‐inventions/clarity), have yet to produce a connectivity map of the human AAN (Teissier et al. 2017).

In this study, we leverage advances in diffusion‐weighted magnetic resonance ex vivo imaging (dMRI) to map, for the first time, the 3‐dimensional connectivity of the AAN in the developing human brain, from the mid‐fetal period to early infancy, with two adult brains representing indices of maturity. Diffusion ex vivo MRI analysis in *postmortem* cohorts of fetuses and infants is specifically challenging due to the lack of available neurotypical samples, the occurring developmental patterns, and the requirement of a highly skilled multidisciplinary team. First, we tested the hypothesis that AAN connectivity can be quantified using diffusion MRI tractography and graph theoretical analysis by performing high‐resolution ex vivo MRI on human brain specimens collected at seven timepoints, ranging from 19 weeks' gestation to 61 years of age We characterized the ontogeny of AAN structural connectivity by identifying network hubs and the composition of local and distant connectivity at each developmental stage. Second, we tested the developmental hypothesis that AAN connectivity becomes increasingly complex, progressing from caudal to rostral brain sites and developing from short‐ to long‐range connectivity during the first year of development. This work is our first step toward the ultimate goal of advancing knowledge of human arousal processes by mapping the ontogeny of human AAN connectivity from early gestation through senescence.

## 2 Materials and Methods

For brain specimen acquisition, informed consent from surrogate decision‐makers was obtained in compliance with standard operating procedures approved by the Mass General Brigham Institutional Review Board (2015B000041, 2019P003875, and 2010P000243). All *postmortem* brains were transferred in accordance with a Material Transfer Agreement (MTA) to the Massachusetts General Hospital Athinoula A. Martinos Center for Biomedical Imaging (MGH Martinos Center) (#2021A012114, 2018A015344) for MRI imaging on a Siemens 3 Tesla (3 T) Tim Trio MRI scanner. Brain acquisition was coordinated via a local autopsy technician and investigator. Prior to postmortem MRI, each specimen was assessed by a neuropathologist following standard institutional protocols. Inclusion criteria of brain specimens in this study were (1) no history of neurological illness (no visible infarct, hemorrhages), (2) no abnormal in vivo brain scan (no visible brain malformation), if applicable (3) normal neurological examination documented by a clinician in the medical record before death, and (4) normal postmortem gross examination of the brain by a neuropathologist. Additional exclusion criteria were cases with impairment of the central nervous system as the main or associated cause of death. In total, we included seven individuals in our study, consisting of three fetal (F1–F3), two infant (N1, N2) and two adult (A1, A2) cases. The protocol details of the dMRI and multi‐echo flash acquisitions and fixation parameters are summarized in Table 2. The fixation procedures used in this study vary slightly between prenatal and postnatal individuals.

Fetal scans: The fetal brains originated from the Allen Institute in Seattle. The brains were immersion fixed for 24 h in 4% periodate‐lysine‐paraformaldehyde with 0.1 M phosphate buffer (PLP). The 19‐ and 22‐week‐old individuals both remained *in cranio* and were surrounded by PLP before the scan, to avoid any artifacts caused by the air‐tissue interface. Additionally, we vacuum sealed each sample in polyethylene bags before imaging to maximize their proximity to the radiofrequency coil components. The 21‐week‐old subject was scanned ex cranio inside a glass tube filled with 1% agarose instead of a liquid medium, to avoid tearing of the sensitive tissue (Kolasinski et al. 2013).

Postnatal infant and adult scans: The postnatal infant brains originated from the University of São Paulo Department of Pathology in São Paulo Brazil. Fixation was performed by placing the specimen in a plastic container and completely covering it with a neutral buffered 10% formalin. Subsequently, we confirmed that no brain surfaces were compressed against the base or sides of the container to avoid any deformations of the tissue. The fixation date and time were documented, and the fixation liquid was changed every week for 1 month to ensure adequate fixation. Infant specimens were fixed for at least 1 month and adult specimens for 3 months.

### 2.1 MRI Acquisition and Post‐Processing

Before MRI imaging, all brain specimens were transferred to a fomblin solution (perfluoropolyether, Ausimont USA Inc., Thorofare, NJ) for at least 1 week to draw out air bubbles trapped in the cortical folds. Imaging in fomblin reduces magnetic susceptibility artifacts. In the coil, all the specimens were secured with padding to reduce motion artifacts induced by vibrations during gradient switching.

*The three fetal scans* were performed using a custom‐built, single‐channel, 2‐turn solenoid coil (6.86 cm inner diameter, 11.68 cm length) with a transmit/receive switch. For the diffusion acquisitions a scan time of 6 h 34 min was required for the 19 and 21wg specimens and 10 h and 50 min for the 22wg specimen. For the multi‐echo flash structural acquisitions the total scan time was 6 min and 53 s per flip angle (in total six were used) (Kolasinski et al. 2013).

*The postnatal scans* of the two infants and two adult brains were acquired using a 32‐channel head coil. dMRI acquisition duration for the infant specimens was 11–19 h. and for the adult specimens was 30 h and 30 min. For the multi‐echo flash structural acquisitions, a total scan time of 1 h 32 min (infants) and 55 min (adults) was required, with 9 min and 16 s per flip angle. In total 10 multi‐echo flash scans of five flip angles (infants) and six flip angles (adults) were used. Figure S1 summarizes the data preprocessing and analytic workflow.

- *dMRI preprocessing*: Tools from the FSL (6.0.4)^1^ and FreeSurfer (Fischl 2012) libraries were used in a customized script to process the diffusion data for probabilistic tractography analysis. For each subject's steady‐state free precession (SSFP) acquisition, we extracted the diffusion gradients and computed b values from estimated T1 values following (McNab et al. 2009). We performed eddy current correction in the anterior–posterior plane to mitigate potential geometric distortions induced by these diffusion imaging artifacts. These artifacts can affect computed maps of diffusion parameters and consequently lead to the misalignment of individual diffusion‐weighted images of an acquired set.
- *sMRI preprocessing*: We performed bias field correction, manual annotation, and 3D reconstruction of 27 subcortical seed regions. To test for cortical connectivity of AAN nodes, we included atlas‐based segmentations of 58 cortical target regions that are consistently identifiable over the fetal, postnatal, and adult cohorts. We used the AAL adult atlas for postnatal and adult cases and the fetal AAL atlas for the fetal cases (Tzourio‐Mazoyer et al. 2002). Table 1 summarizes the defined subcortical seeds and cortical target regions. The atlas labels were propagated from the individual's sMRI space to the individual's dMRI space via a block‐matching method for global registration (Modat et al. 2014). All aligned images and segmentations were visually inspected to confirm accurate alignment.
- *Tractography*: Probabilistic tractography In order to obtain statistical robustness for our structural connectivity estimates between two regions of interest, first voxel‐wise distributions of diffusion directions were estimated using FSL bedpostx. Subsequently, posterior streamline distributions were derived by repeatedly sampling streamlines with FSL probtrackx2 (Behrens et al. 2007; Boly et al. 2017) to derive diffusion direction estimations weighted by the number of voxels within each region using the tools' default parameters (5000 seeds per voxel, 2000 steps per seed, 0.5 mm step size, 0.2 curvature threshold), as we had previously done in (Edlow et al. 2024). We relied on the 27 subcortical labels as seed and target and the 58 cortical labels as target regions. The fitting of the probabilistic diffusion model utilized bedpostx (Jbabdi et al. 2012) and white matter brain masks were used to spatially restrict the location of fiber bundles. Probabilistic tractography enables the computation of the connectivity probability between two regions, which may be associated with the distance between these (Edlow et al. 2023). Since we do not know if this relation is of a linear or exponential nature and if a single distance correction method would account for the fiber bundle tracts' dependency on multiple variables (number of other bundles that intersect the bundle of interest along its course, the number of voxels in the seed and target region, and the bundle‘s geometry), we decided to not perform correction for the path distribution using pathway‐length. We processed each label independently, given that there is known asymmetry in the development of the left and right hemispheres in the neonatal brain, which has an effect on structural connectivity computations and graph‐based analysis (Ratnarajah et al. 2013).
  To facilitate visualization of AAN pathways, we performed complementary deterministic tractography analyses using Diffusion Tool Kit and TrackVis (Version 0.6.4.1). dMRI data were reconstructed using the orientation distribution function model and fiber tracking was performed across the entirety of each brain specimen with an angle threshold of 60°, consistent with prior studies of the adult human AAN (Edlow et al. 2012). A binary brain mask was used to terminate tracts extending beyond brain tissue, as reported previously (Kolasinski et al. 2013; Takahashi et al. 2011). The resulting whole‐brain tractography outputs were passed through a spline filter. The brainstem nodes of the AAN formed the anatomical basis for the deterministic tractography analysis. They were used to seed white matter tractography reconstruction.
- *Graph construction and analysis*: The output of the probabilistic tractography for each node pair was entered into a connectivity matrix, which was normalized by the total number of streamlines used by probabilistic tractography, resulting in structural connectivity values ranging from 0.0 (no connectivity) to 1.0 (strong connectivity).

**TABLE 1:** Annotated regions in the brainstem, diencephalic and cortical brain areas.

Twenty seven brain seed regions of the ascending arousal network		
Abbreviation	Description	System/number of ROIs
VTA	Ventral tegmental area	Dopaminergic, Limbic/1
BF (L + R)	Basal Forebrain	Cholinergic/6
LDTg (L + R)	Laterodorsal tegmental nucleus	
PTg (L + R)	Pedunculotegmental nucleus	
LC (L + R)	Locus coeruleus	Noradrenergic, Cardio‐Respiratory/2
DR	Dorsal raphe	Serotonergic 1
MnR	Median raphe	Serotonergic, Cardio‐Respiratory/1
CR	Caudal raphe	Serotonergic, Reticular core, Cardio—
PGCL (L + R)	Paragigantocellularis lateralis	Respiratory/3
VC (L + R)	Vagal complex	Autonomic nervous system, Cardio‐Respiratory/2
mRt (L + R)	mesencephalic Reticulation formation	Reticular core/4
PnO (L + R)	Pontine reticular formation (pontis oralis)	
PBC (L + R)	Parabrachial complex	Reticular core, Glutamatergic, Cardio‐Respiratory, Limbic/2
Th (L + R)	Thalamus	Diencephalon/2
HY (L + R)	Hypothalamus	Limbic forebrain, Autonomic nervous system/2
PAG	Periaqueductal gray	Limbic brainstem, Enkephalin/1

Fifty‐eight cortical regions defined by the AAL Atlas (Tzourio‐Mazoyer et al. 2002)		
Abbreviation	Description	Cortical region/number of ROIs
Precentral (L + R)	Precentral gyrus	Central Region/2
Postcentral (L + R)	Postcentral gyrus	Central Region/2
Frontal_Sup (L + R)	Superior frontal gyrus, dorsolateral	Frontal lobe‐lateral surface/6
Frontal_Inf_Tri (L + R)	Inferior frontal gyrus triangular part	
Frontal_Mid (L + R)	Middle frontal gyrus	
Frontal_Med_Orb (L + R)	Superior frontal gyrus, medial	Frontal lobe‐medial surface/6
Supp_Motor_Area (L + R)	Supplementary motor area	
Paracentral_Lobule (L + R)	Paracentral lobule	
Frontal_Sup_Orb (L + R)	Superior frontal gyrus, orbital part	Frontal lobe‐orbital surface/10
Frontal_Sup_Medial (L + R)	Superior frontal gyrus, medial part	
Frontal_Mid_Orb (L + R)	Middle frontal gyrus, orbital part	
Frontal_Inf_Orb (L + R)	Inferior frontal gyrus, orbital part	
Olfactory (L + R)	Olfactory cortex	
Temporal_Sup (L + R)	Superior temporal gyrus	Temporal lobe‐lateral surface/8
Temporal_Mid (L + R)	Middle temporal gyrus	
Temporal_Inf (L + R)	Inferior temporal gyrus	
Heschl (L + R)	Heschl gyri (Primary auditory system)	
Temporal_Pole_Mid (L + R) Temporal_Pole_Sup (L + R)		Temporal lobe/4
Parietal_Sup (L + R)	Superior parietal gyrus	Parietal lobe‐lateral surface/4
Parietal_Inf (L + R)	Inferior parietal—supramarginal and angular gyri	
Precuneus (L + R)	Precuneus	Parietal lobe‐medial surface/2
Occipital_Sup (L + R)	Superior occipital gyrus	Occipital lobe‐lateral surface/6
Occipital_Inf (L + R)	Inferior occipital gyrus	
Occipital_Mid (L + R)	Middle occipital gyrus	
Cuneus (L + R)	Cuneus	Occipital lobe‐medial and inferior surfaces/4
Calcarine (L + R)	Calcarine (Primary sensory system)	
Lingual (L + R)	Lingual Gyrus	Occipital lobe‐medial and inferior surfaces/2
Insula (L + R)	Circular sulcus of the insula	Insular lobe/2

*Note:* ‘R’ represents the annotation on the right hemisphere and ‘L’ on the left.

**TABLE 2:** Demographics, procedures and sequences for postmortem MRI.

ID	AD	Sex	PMI (h)	F	State	Sequence
F1	19 wg	M	< 21	24 h	In cranio	sMRI (3 T): TR = 25 ms, *α* = [10°, 20°, 30°, 40°, 50°, 60°]; TE = [2.93, 5.83, 8.93, 12.23, 15.73, 19.43] ms; 500 μm isotropic dMRI (3 T): TR = 24.5 ms, *α* = 60°, TE = 18.76 ms, 400 μm isotropic, 44 directions (*b* = ~730 s/mm^2^) with 4 *b* = 0 images
F2	21 wg	M	< 21	24 h	Ex cranio	
F3	22 wg	F	< 21	24 h	In cranio	
N1	1 months	M	6	49 days	Ex cranio	sMRI (7 T): TR = 40 ms; *α* = [10°, 20°, 30°, 40°, 50°]; TE = [4.96, 12.28] ms; 550 μm isotropic dMRI (3 T): TR = 29 ms, *α* = 35°, TE = 24.5 ms, 700 μm isotropic, 80 directions (*b* = ~3782 s/mm^2^) with 10 *b* = 0 images
N2	2 months	M	3.5	28 days	Ex cranio	sMRI (3 T): TR = 23 ms; *α* = [5°, 10°, 15°, 20°, 25°, 30°]; TE = [2.64, 4.48, 6.32, 8.16, 10.00, 11.84, 13.68, 15.52, 17.36, 19.20] ms; 500 μm isotropic dMRI (3 T): TR = 29 ms, *α* = 35°, TE = 24.5 ms, 700 μm isotropic, 90 directions (*b* = ~3782 s/mm^2^) with 15 *b* = 0 images
A1	60 years	F	24	> 90 days	Ex cranio	sMRI (3 T): TR = 23 ms; *α* = [5°, 10°, 15°, 20°, 25°, 30°]; TE = [2.64, 4.48, 6.32, 8.16, 10.00, 11.84, 13.68, 15.52, 17.36, 19.20] ms; 500 μm isotropic dMRI (3 T): TR = 28.87 ms, *α* = 35°, TE = 24.44 ms, 750 μm isotropic, 90 directions (*b* = ~3773 s/mm^2^) with 12 *b* = 0 images
A2	61 years	F	72	> 90 days	Ex cranio	

Abbreviations: AD, age at death; dMRI, diffusion weighted MRI; F, fixation time; PMI, postmortem interval; sMRI, structural MRI; wg, week of gestation.

### 2.2 Identification of Candidate Ascending Arousal Network Seed Regions

The brainstem neurotransmitter systems of arousal form defined nuclei and circuits that are specified in Table 1 and Figure 3. We use these nuclei and circuits extrapolated from human cytoarchitectonic and chemoarchitectonic studies in the literature as anatomic surrogates for the transmitter pathways of the primary specific neurotransmitter, as in previous studies (Edlow et al. 2016; Edlow et al. 2023; Edlow et al. 2012). For the AAN analyses, we identified 27 seed ROIs that cover the brainstem, hypothalamus, thalamus, and basal forebrain nuclei. For the identification of diffuse projections between cortical regions and the AAN in the brainstem, we additionally selected 58 relevant cortical regions from the AAL‐atlas (Tzourio‐Mazoyer et al. 2002), with the requirement to be identifiable in all specimens (see Table 1).

The neurochemical systems play an arousal‐promoting role including the triggering of fast cortical activities during the wakefulness state (Scammell et al. 2017). Neurons of these systems can be categorized as follows (see also Table 1 and Figure 3 for a summary):

- Noradrenergic: lying in the locus coeruleus (LC).
- Serotonergic: lying in the dorsal (DR) and median raphe (MnR) nuclei.
- Histaminergic: lying in the tuberomammillary nucleus.
- Dopaminergic: lying in the ventral tegmental area (VTA).
- Glutamatergic: lying in the parabrachial complex (PBC).
- Cholinergic: lying in the pedunculopontine (PTg), laterodorsal tegmental nucleus (LDTg) and basal forebrain (BF).
  Ascending pathways originating from the midbrain's paramedian region are wake‐promoting and reach the thalamus via the dorsal pathway (enabling consciousness by thalamic signaling like motor responses, cognition, sensation) and the hypothalamus, basal forebrain and cortex via a ventral pathway (enabling the behavioral state of wakefulness) (Scammell et al. 2017).

### 2.3 Probabilistic Tractography and Structural Connectivity Estimations

To compute structural connectivity, local probability density functions (PDFs) from the simple partial volume model were used. The spatial PDFs were computed in the pre‐processing, to represent the complexity of fiber structures by uncertainty estimates along principal diffusion directions. Therefore, a spatial PDF describing the probability that a seed region A is connected to a target region B is estimated by generating an extensive number (e.g., 5000) of *single probabilistic streamlines* in the following way: Starting from a seed region A at every local point of the stream a random sample of fiber orientation is drawn from the distribution of fiber orientations and used as the direction to continue the stream to the next local point within a defined distance *s*. This procedure is performed until a stopping criterion is reached.

By the discretization of the resulting distribution, we obtained a connectivity probability (CP) value of a seed region A (incorporating all of its voxels) being connected to region B, by dividing the number of connecting probabilistic streamlines by the total number of probabilistic streamlines launched from A (Snider et al. 2019).

(1) $${\mathrm{CP}}_{A\to B}=\frac{\left[\text{Number of tracts connecting}\;A\;\text{to}\;B\right]\;}{\left[\text{Number of tracts launched from}\;A\right]}$$

In large‐scale brain networks, streamline likelihoods tend to decrease as a function of path length due to the propagation of uncertainty along the streamline, resulting in distance false negatives (Chang et al. 2022; Morris et al. 2008) at long distances. To counteract this effect, we additionally computed the log of the connectivity probability values for visualizing and interpreting the connectivity matrices.

### 2.4 Graph‐Based Representations for Structural Connectivity Analysis

Graph‐based measures have been widely used to characterize brain network properties (Sporns 2010; Sporns et al. 2007; Yuan et al. 2015). The complete network of neuronal connections in the human brain is labeled the *connectome* (Oldham and Fornito 2019; Sporns et al. 2007). Analyses of the *connectome* define the brain as a graph, consisting of nodes connected by so‐called edges (Bullmore and Sporns 2009; Fornito et al. 2015; Oldham and Fornito 2019; Rubinov and Sporns 2010; Sporns 2010) that represent either a measure of structural or functional connectivity.

In a graph, *nodes* represent specialized neuronal populations or nuclei in brain regions of interest, here denoted as R, defined by parcellations, segmentations, or atlases (Arslan et al. 2018; Fornito et al. 2015; Oldham and Fornito 2019; Wig et al. 2011). The *edges* between nodes represent connections between brain regions. Each connection between a source region $R_{i}$ and a target region $R_{j}$ varies in its strength, encoded by the edge weight $w_{\mathrm{ij}}$. This edge weight can be denoted as the connectivity probability ${\mathrm{CP}}_{R_{i}\to R_{j}}$ between the two brain regions $R_{i}$ and $R_{j}$.

A graph can mathematically be represented as a matrix $C\left(i,j\right)$ of size *N* × *M*. The rows $i=\left\{1,..,N\right\}$ represent the seed nodes and the columns the target nodes $j=\left\{1,..,M\right\}$. The values of the structural connectivity matrix $C\left(i,j\right)$ are computed following Equation (2), which encodes the *CP* for each pair of brain ROIs.

(2) $$C\left(i,j\right)={\mathrm{CP}}_{R_{i}\to R_{j}}=\frac{\left[\text{Number of tracts connecting}\;R_{i}\;\text{to}\;R_{j}\right]\;}{\left[\text{Number of tracts launched from}\;R_{i}\right]},i=\left\{1,..,N\right\},j=\left\{1,..,M\right\}$$

In this work two regions $R_{i}$ and $R_{j}$ are considered to be structurally connected, if $C\left(i,j\right)$ is larger than a defined threshold. This information is encoded in an adjacency matrix $A\left(i,j\right)$, which is obtained by binarizing $C\left(i,j\right)$ using the threshold th. $A\left(i,j\right)$ is 1 if nodes *i* and *j* are connected and 0 otherwise.

Once a network has been constructed, the measures of graph theory (Barabási 2013; Newman 2010) can be used to quantify different properties of the network (Oldham and Fornito 2019). Within complex and intricate networks, structural nodes are thought to possess a relatively large number of connections that are unevenly distributed, defining them as putative network hubs (van den Heuvel and Sporns 2013).

According to (Oldham and Fornito 2019) “brain hubs facilitate the integration of functionally specialized and anatomically disparate neural systems, a role supported by their tendency to form long‐range connections, and their topological position within the brain, which suggests that they mediate a large fraction of signal traffic”. Hubs are implicated in many neurological disorders of arousal and homeostasis, including coma, sleep‐disordered breathing, adult obstructive sleep apnea, and the sudden infant death syndrome (Crossley et al. 2014; Fornito et al. 2015).

### 2.5 Hub Rank Computation

Here, we assess potential network hubs by combining the following three local graph‐based measures (Borgatti and Everett 2006; Fornito et al. 2015; Sporns et al. 2007):

- Degree *D*_*i*_: The degree of a node denotes the number of edges connected to this node. In Figure S2 on the left, for example, the degree of the red node is three and is computed by the sum of connected edges (visualized in red) to this node:

(3) $$D_{i}=\sum_{j=1}^{N}A\left(i,j\right)$$

- Local clustering coefficient *C*_*i*_: The clustering coefficient of a node *i* is the number of triangles surrounding it in relation to the maximum number of potential connections to node *i* (see Equation 4). An example of a triangle structure in a graph is visualized in green in Figure S2. A cluster can be defined as a subset of nodes with a high number of edges between these. A local cluster coefficient close to 1 (yellow node Figure S2) means the node and its connections are more likely to form a cluster, while nodes with a value close to 0 (middle blue node Figure S2) are more likely to form hubs.

(4) $$C_{i}=\frac{\left[\text{Number ofconnected triangles including node}\;i\;\right]\;}{\text{Maximum number of potential connections to node}\;i\;}=\frac{\left[\sum_{1\leq j<l\leq N}A\left(i,j\right)A\left(i,l\right)A\left(j,l\right)\;\right]\;}{\left[D_{i}\;\left(D_{i}-1\right)/2\right]\;}$$

- Betweenness centrality *B*_*i*_: This measure denotes the fraction of all shortest paths, which contain node *i*. A high value of betweenness centrality can be interpreted as a node that participates in a large number of shortest paths between two nodes (see Equation 5) (Kintali 2008). An example of a shortest path between two nodes, *x* and *y*, is visualized in purple in Figure S2 on the left.

(5) $$B_{i}=\sum_{x\neq i\neq y}\frac{\left[\text{Number ofshortest paths between}\;x\;\text{and}\;y\;\text{passing through node}\;i\;\right]\;}{\text{Number ofshortest paths between}\;x\;\text{and}\;y\;}=\sum_{x\neq i\neq y}\frac{\left[P_{\mathrm{xy}}\left(i\right)\right]\;}{P_{\mathrm{xy}}\;}$$

The *hub rank of a region* is determined by the mean of the ranks of all graph‐based measures associated with that region (Yuan et al. 2015). In our case, every node (seed brain ROI) was ranked separately for every graph‐based measure between 1 and N, where N, the highest rank, was set to 27 (total number of seed ROIs). Regions with the lowest clustering coefficient, highest betweenness and highest degree measure receive the highest hub rank.

### 2.6 Long‐Range and Short‐Range Structural Connectivity Computation

In addition to hub rank computations, we introduce long‐range and short‐range connectivity as a score to assess developmental differences. These measures have been widely used for assessing functional connectivity in adults (Sepulcre et al. 2010) and children (Licandro et al. 2016), as well as for structural connectivity analysis (Ouyang et al. 2015; Ouyang et al. 2017). Here, we define short‐range structural connectivity as fiber tracts that are shorter than a specified physical distance ${\mathrm{th}}_{Q}$ and long‐range connectivity as fiber tracts that are longer (Ouyang et al. 2017).

As a first step, we computed the distance matrix $T\left(i,j\right)$ to encode distances between the observed regions. For every region, we obtained a 3D reconstruction of the annotation and computed its barycenter and the Euclidean distance between every node's barycenter coordinates. As a second step, for every node, we consider the distances only to connecting nodes for graph‐based computations and categorize distances as long‐range, if these are above a threshold ${\mathrm{th}}_{Q}$, and short‐range if these are below or equal. For the graph‐based analysis we compute the number of short‐range $S_{\mathrm{SR}}\left(i\right)$ and long‐range $S_{\mathrm{LR}}\left(i\right)$ (see Equations (6) and (7)).

(6) $$S_{\mathrm{SR}}\left(i\right)=\left[\text{Number of}\;\mathrm{SR}\;\text{connection of node}\;i\;\right]=\sum_{j=1}^{N}\left(\left[T\left(i,j\right)\times A\left(i,j\right)\right]\leq {\mathrm{th}}_{Q}\right)$$

(7) $$S_{\mathrm{LR}}\left(i\right)=\left[\text{Number of}\;\mathrm{LR}\;\text{connection of node}\;i\;\right]=\sum_{j=1}^{N}\left(\left[T\left(i,j\right)\times A\left(i,j\right)\right]>{\mathrm{th}}_{Q}\right)$$

## 3 Results

**FIGURE 2:**
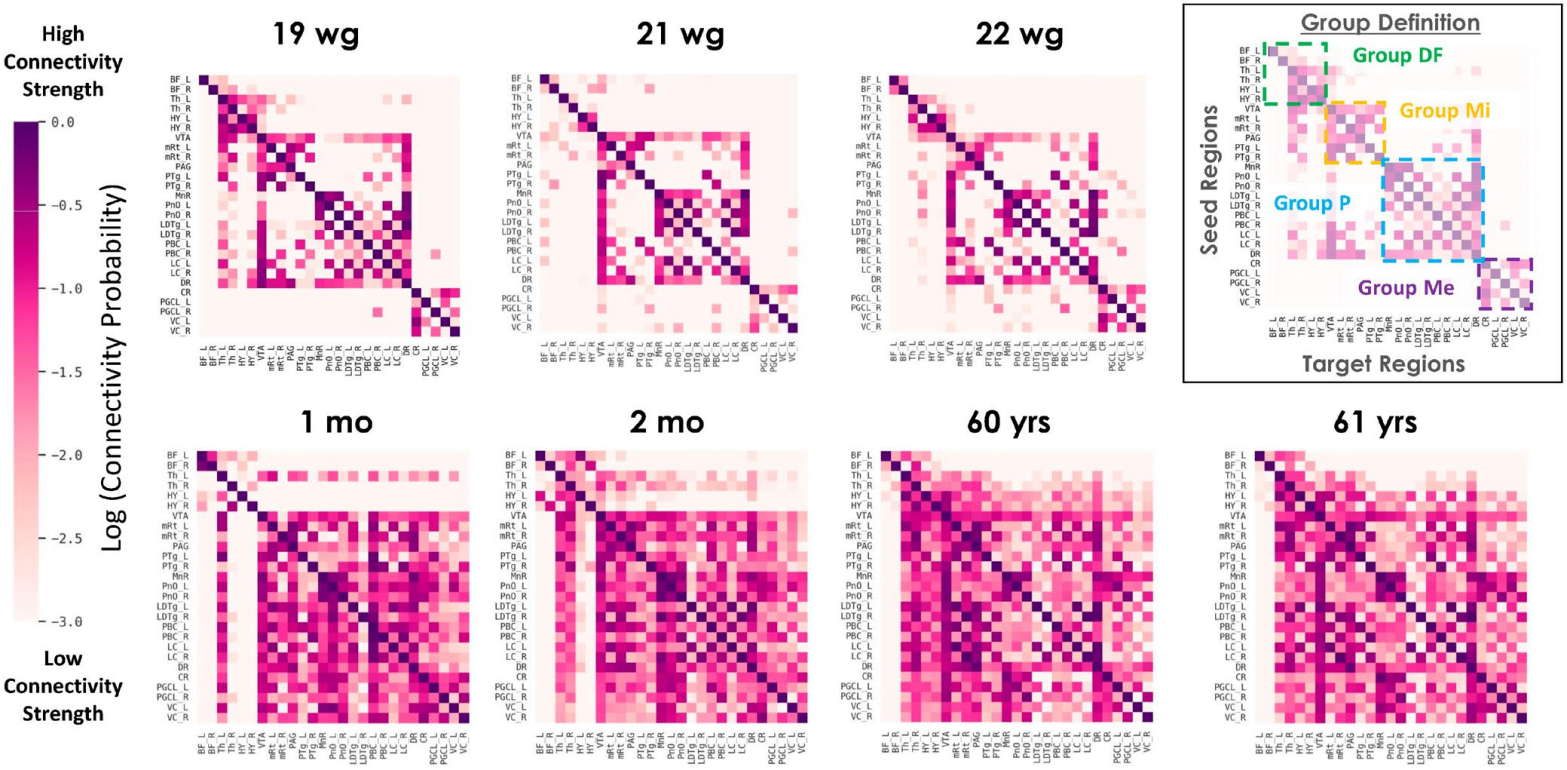
Age‐dependent variation of structural connectivity. Displayed are matrices encoding connectivity strength (represented as log CP) between AAN regions of interest, of individuals from the fetal period of 19, 21, and 22 weeks of gestation (wg), from the postnatal period of 1 and 2 months (mo), and the adult period of 60 and 61 years. The 27 seed regions are ordered from rostral to caudal. On the top right, the identified connectivity groups in the fetal cases (Group DF, Group Mi, Group P, and Group Me) are visualized overlaid onto the 19wg fetal case. Group DF, Diencephalon/forebrain cluster; Group P, Rostral brainstem—pons cluster; Group Mi, Rostral brainstem—midbrain and pontomesencephalic junction cluster; Group Me, medulla cluster connections.

We first analyzed the connectivity probability (CP)—a measure of structural connectivity derived from dMRI probabilistic tractography data—for 27 regions of interest (ROIs) in the AAN (see Table 1) (Snider et al. 2019). CP values for each brain specimen are encoded in a connectivity matrix and visualized in Figure 2 in log scale, with ROIs ordered from the rostral (top) to the caudal (bottom) axis of the human brainstem. We observed a doubling of connections (degree) with CP > 0.5 per seed region from the fetal to postnatal period. At age 19wg, we found that connections are first established within four groups of anatomically adjacent AAN regions: medulla *Me*, rostral‐brainstem‐pons *P*, rostral‐brainstem‐midbrain *Mi* and forebrain diencephalon DF (Thalamus—Th, Hypothalamus—HY, Basal Forebrain—BF) (see Figure 2, Table T1). Connections between Groups P and Mi are already established via DR and VTA in the fetal period (19wg—21 wg), while long‐range connectivity among the remaining clusters emerges postnatally. Further, we found increasing connectivity strength from all caudal to rostral brainstem regions correlating with increasing age, as well as the timing and synchronization of fetal‐to‐postnatal myelination from caudal to rostral levels of the neuroaxis (Kinney et al. 1988; Kinney and Volpe 2018). In the adults, the connectivity patterns appeared relatively stable.

**FIGURE 3:**
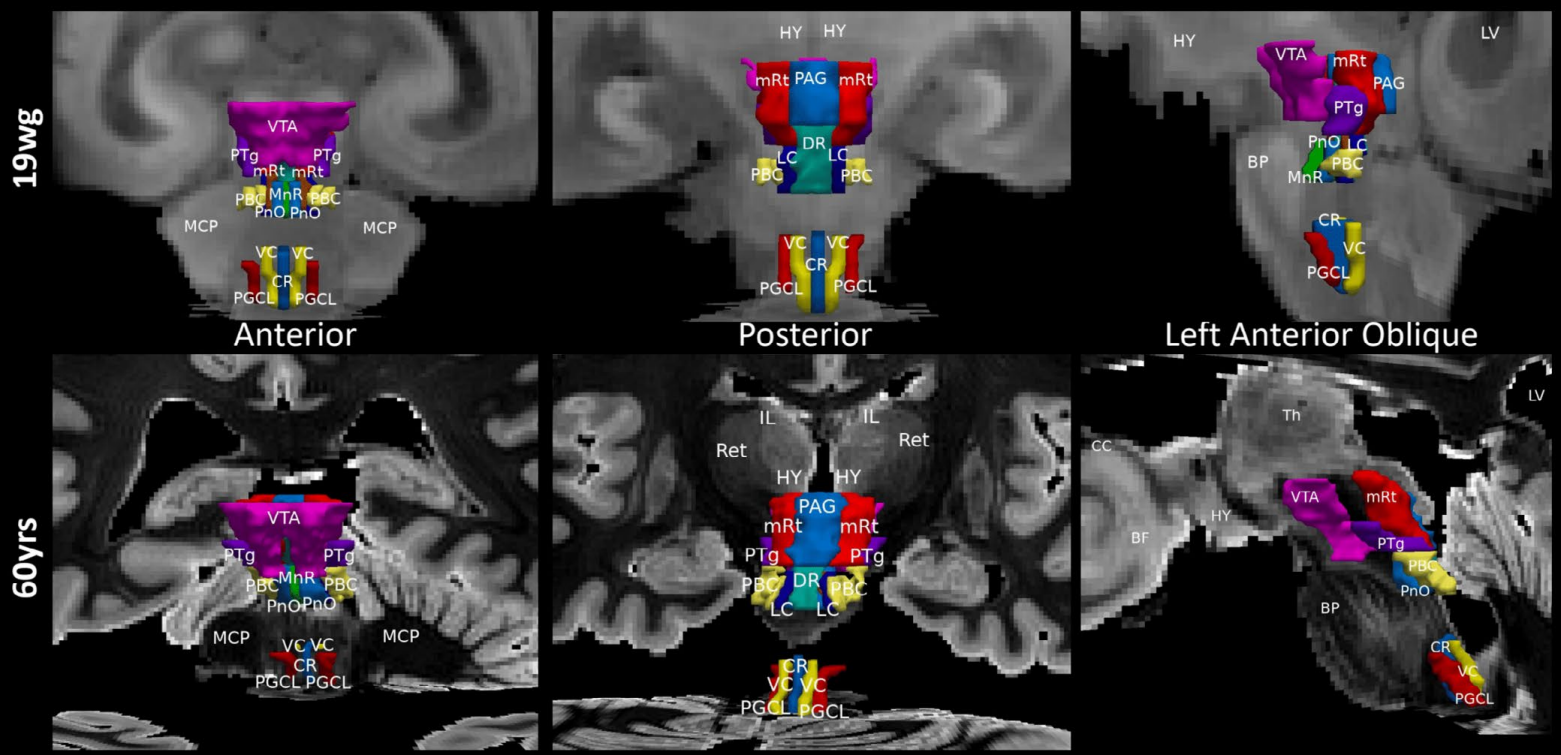
Manually annotated ROIs of the AAN in the brainstem for 19wg and 60 years subjects. CC denotes the corpus callosum, LV lateral ventricle, (IL) intralaminar nuclei, (MCP) middle cerebellar peduncles, (BP) basis pons, and (Ret) reticular nuclei. The abbreviations of the 27 seed regions of the AAN are summarized in Table 1.

**FIGURE 4:**
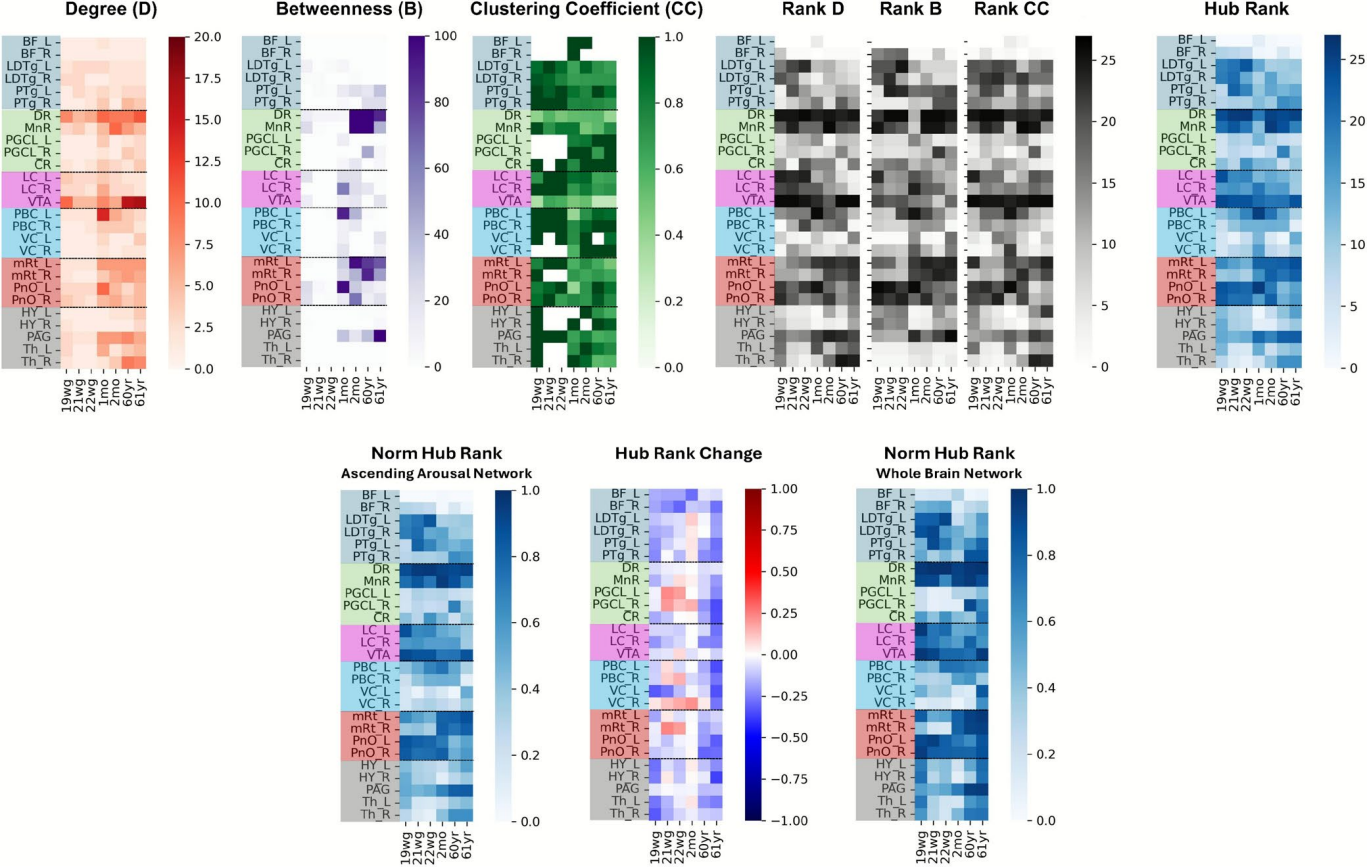
Graph‐based longitudinal analysis of ascending arousal network regions. (Top row) Graph‐based measures of degree (D, red), betweenness (B, purple), clustering coefficient (CC, green), corresponding rank computations (grey), and resulting hub ranks (blue) across age, using a connectivity probability threshold of 0.5 for computation. Ranks have values between 1 and 27 (the total number of target regions). Regions of interest (y‐axis) are grouped by ACh nuclei (blue), Serotonin nuclei (green), Dopamine/Noradrenaline nuclei (pink), respiratory nuclei (yellow), reticular core (red), thalamic nuclei and PAG (gray). (Bottom row) Comparison of Relative Hub Ranks of ROIs within the AAN (27 Regions, left) and the Whole Brain Network (85 Regions: 27 AAN Seed Regions +58 Cortical Regions, right). To perform this comparison, hub rank value ranges between 0 and 27 (for the 27 AAN target regions), and 0 and 85 (for the 85 whole brain network target regions) were normalized to fall within the range 0 and 1. The change of Relative Hub Rank between 27 and 85 regions is visualized in the middle. Blue values indicate higher importance as a hub in the context of the whole brain network, while red values indicate higher importance as a hub within the brainstem AAN. Regions of interest (y‐axis) are grouped by ACh nuclei (blue), Serotonin nuclei (green), Dopamine/Noradrenaline nuclei (pink), respiratory nuclei (yellow), reticular core (red), thalamic nuclei and PAG (gray).

**FIGURE 5:**
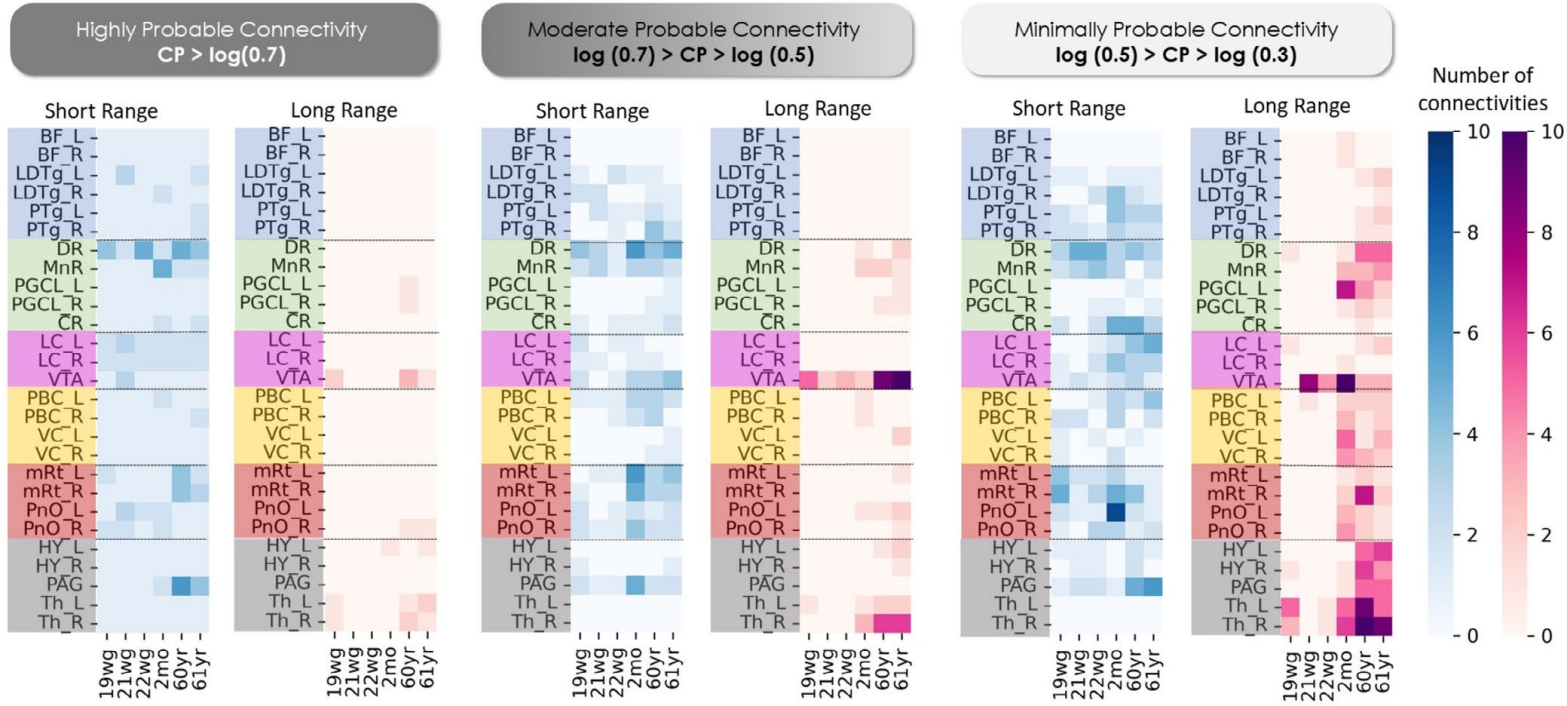
Visualization of the number of short range (SR) and long range (LR) structural connection approximations over age. This visualization includes connectivity estimates of different connectivity probability value ranges: Every subfigure has on the y‐axis the regions of interest grouped by ACh nuclei (blue), Serotonin nuclei (green), Dopamine/Noradrenaline nuclei (pink), respiratory nuclei (yellow), reticular core (red), thalamic nuclei and PAG (gray) and on the x‐axis the age. Blue colors encode the number of SR connectivities within a seed region for a specific age, while purple colors the number of LR connectivities. Columns 1 and 2 visualize short range and long range of highly probable connectivity (> 0.7) columns 3 and 4 of moderate probability (0.7 > CP > 0.5) and columns 5 and 6 of slightly probable connectivities (0.5 > CP > 0.3).

**FIGURE 6:**
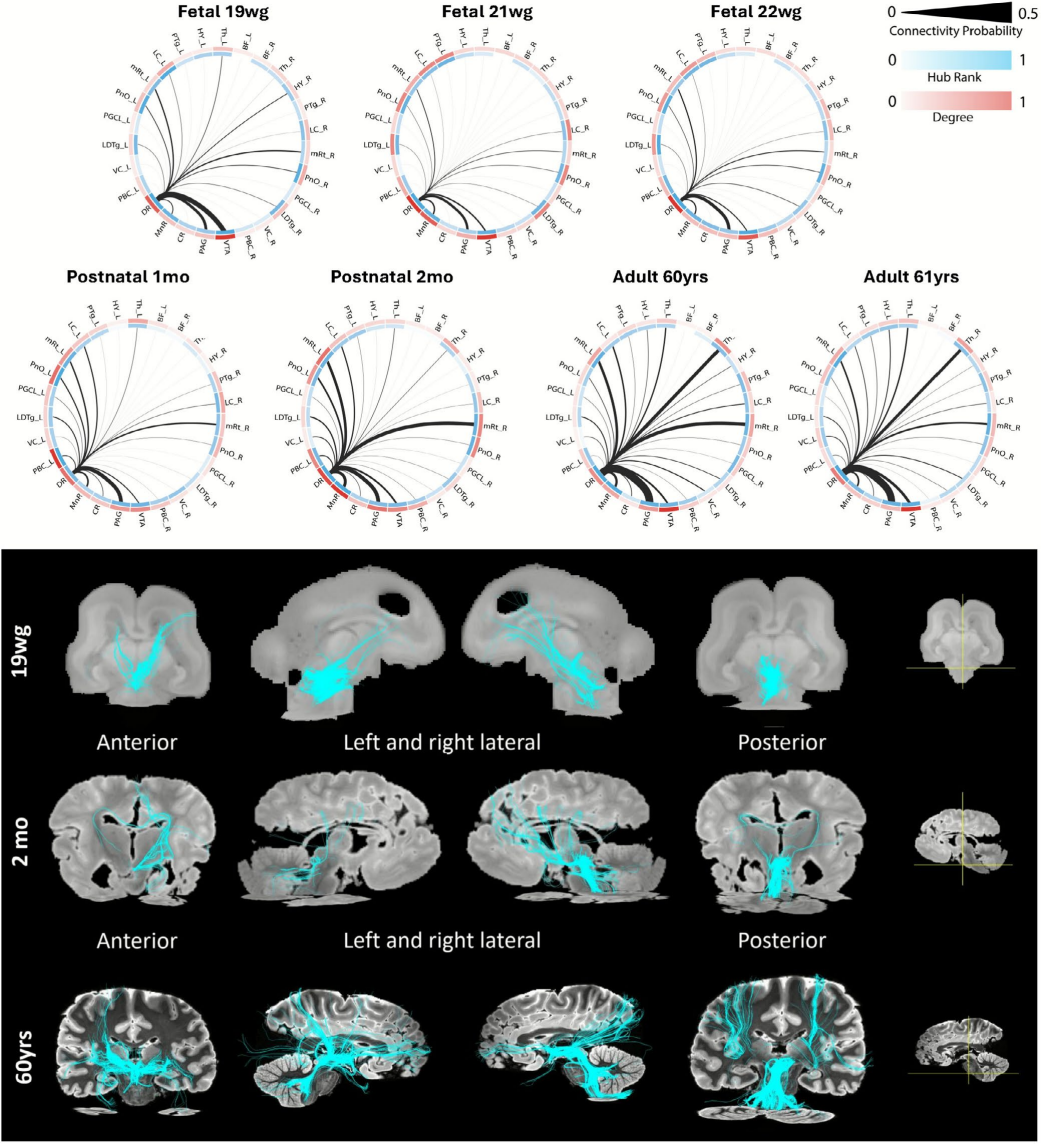
Longitudinal Dorsal Raphe connectivity analysis. (Top panel) Connectograms using the dorsal raphe (DR) nucleus as seed to visualize the corresponding connectivity probability and graph‐based measure changes across age. The outer circle visualizes the degree value for every node (white‐red), and the inner circle the hub rank (white‐blue). The link thickness encodes the connectivity probability. (Bottom panel) DR seed region and corresponding deterministic tractography visualizations. The fetal and postnatal brain images were scaled to approximately match the adult samples for easier qualitative comparison.

**FIGURE 7:**
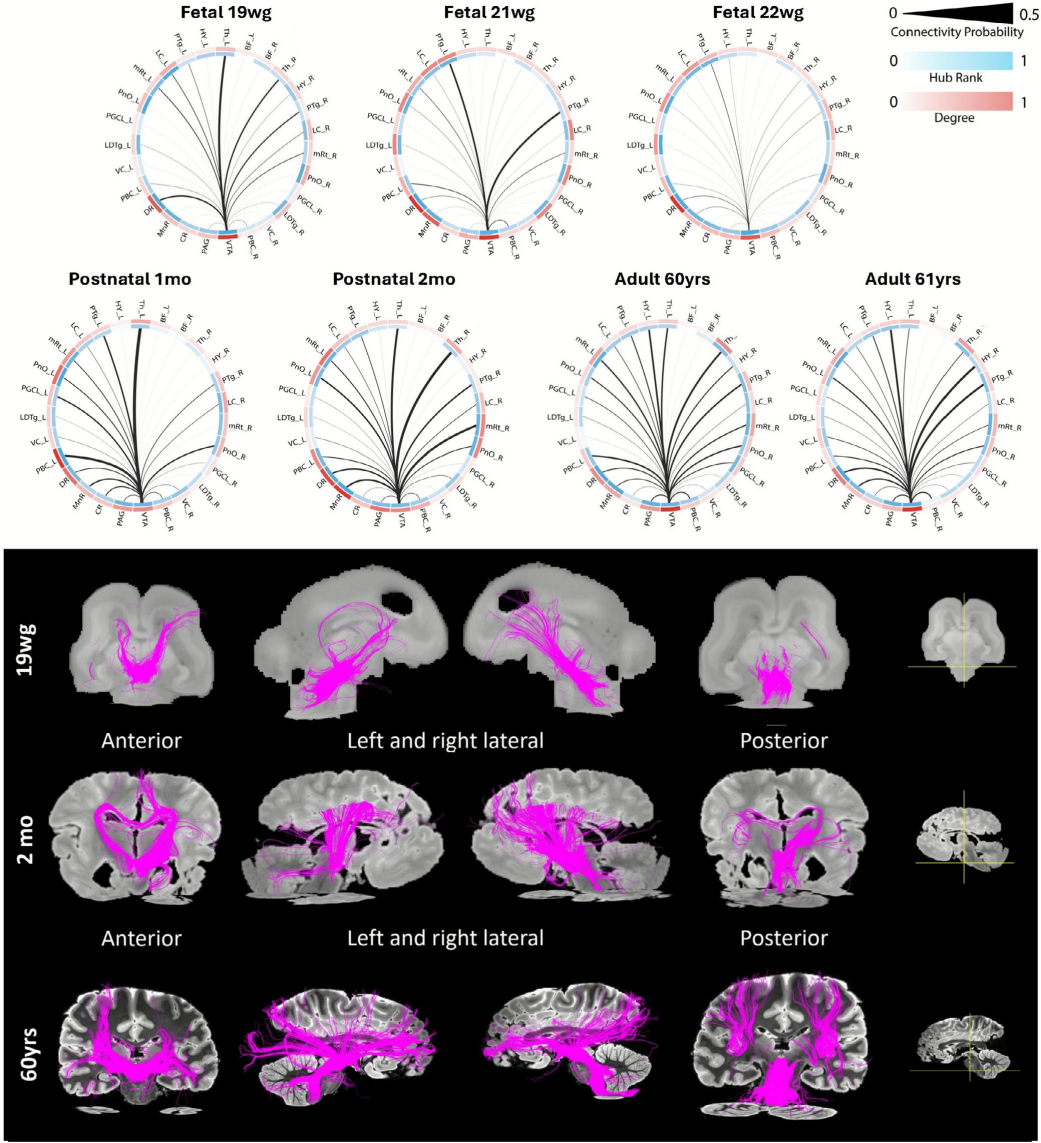
Longitudinal ventral tegmental area connectivity analysis. (Top panel) connectograms of the ventral tegmental area (VTA) to 27 target regions across age. The outer circle visualizes the degree value for every node (white‐red), and the inner circle the hub rank (white‐blue). The link thickness encodes the connectivity probability. (Bottom panel) VTA seed region and corresponding deterministic tractography visualizations. The fetal and postnatal brain images were scaled to approximately match the adult samples for easier qualitative comparison.

**FIGURE 8:**
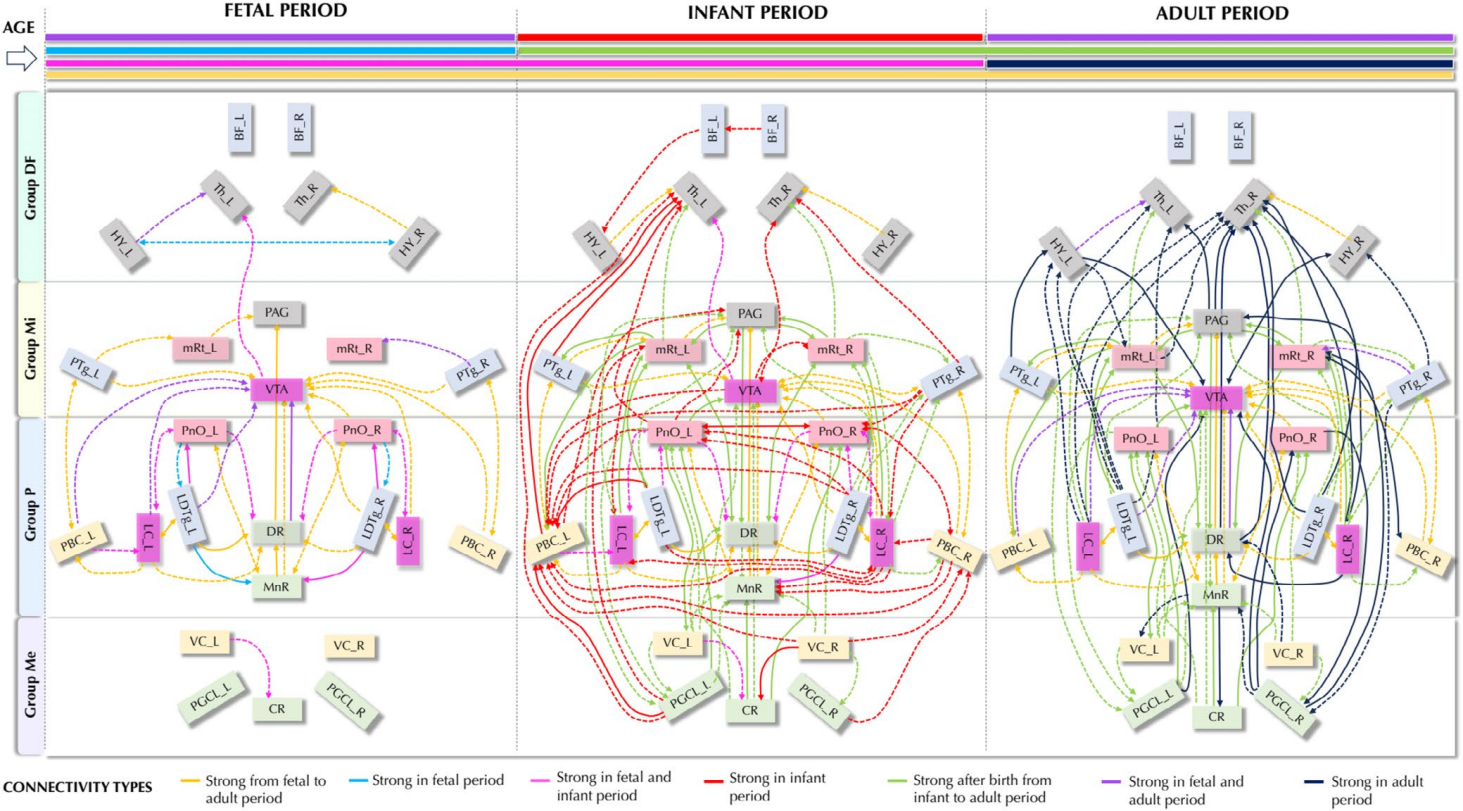
Sequence graph diagram of the developing connectivity strength across age ranges. Moderate‐to‐strong connections (CP > 0.2) are displayed in the diagram. Solid line: all individuals within the age group show this connectivity. Dotted lines: at least one subject in the age group shows the relevant strong connection. For example: a dotted yellow line means at least one fetus, one infant, and one adult showed this connection; a dotted pink line means that at least a fetus and an infant showed this strong connection. The direction of each arrow indicates the direction from seed to target region of the tractography estimates. Group DF, Diencephalon/forebrain cluster; Group P, Rostral brainstem—pons cluster; Group Mi, Rostral brainstem—midbrain and pontomesencephalic junction cluster; Group Me, medulla cluster connections.

### 3.1 Assessment of Structural Connectivity Dynamics Using Graph‐Based Measures

For assessing developmental patterns of structural brain connectivity quantitatively, we computed the regional network measures *degree D*, *betweenness B*, and *clustering coefficient CC* to derive the *hub rank* for 27 seed ROIs in the AAN (see Table 1 for ROIs and abbreviations), only considering connections with a CP>0.5. The results of computed regional network measures are visualized in Figure 4. The *degree* ***D*** of a region can be interpreted as the number of regions connected to this ROI. For all age groups, including the earliest (19wg), the dorsal raphe (DR) and ventral tegmental area (VTA) were connected to more than 40% (*degree* 10) of the 27 AAN seed regions. We also observed increasing ***D*** measures among the mesencephalic reticulation formation (mRt), pontis oralis (PnO), parabrachial complex (PBC), and periaqueductal gray (PAG), particularly between the pre‐ and postnatal brains. A high value of *betweenness* ***B*** can be interpreted as a node that participates in many shortest paths between two nodes. The *betweenness* increased from pre‐ to postnatal cases (B value increase by around 40+) in the network containing the regions DR, median raphe (MnR), mRt, or PAG. For the pontine nodes locus coeruleus (LC), PBC, and PnO, the highest ***B*** values occurred in the age group 1–2 months, while for the hypothalamus (HY), thalamus (Th), basal forebrain (BF), paragigantocellularis lateralis (PGCL), caudal raphe (CR) and vagal complex (VC) the lowest *B* measures (*B* values < 20) occurred over all ages. The *CC* represents the fraction of connection triangles around a region that is, the fraction of a seed region's neighbors that are neighbors of each other. The DR and VTA show the lowest values < 0.4 over all age ranges. A strong decrease between fetal and postnatal cases is visible in PnO, pedunculotegmental nucleus (PTg), laterodorsal tegmental nucleus (LDTg), PBC, LC, and PAG, in contrast to an increase in PGCL, Th, and VC.

### 3.2 Hub Rank Analysis Within the AAN and Whole Brain Network

We focused the graph‐based analysis on hub rank because it provides information about the presumed functional importance of a region within a defined network. Hubs are implicated in many neurological disorders of arousal and homeostasis, including coma, sleep‐disordered breathing, adult obstructive sleep apnea, and sudden infant death syndrome (Crossley et al. 2014; Fornito et al. 2015; Oldham and Fornito 2019). Figure 4 highlights the hub ranks for each of the 27 ROIs. Notably, the DR and VTA show the highest hub ranks (dark blue) for every age group, while BF and VC have the lowest. An increase in hub rank with increasing age is visible in the mRt, PAG, and PBC, while a decrease in rank from fetuses to infants is observed in HY, LC, LDTg, and in PnO between younger individuals and adults. Hub rank trajectories thus vary depending on the time period studied. In particular, in the fetal period, low hub ranks in PBC, VC, PGCL, and PAG predominate.

We extended this hub rank analysis by incorporating 58 additional cortical target regions (in total 85 ROIs, details can be found in Table 1) to identify whether specific brainstem regions also remain highly ranked hubs for cortical projections or only obtain high ranks within the subcortical AAN (Figure 4). Within the AAN, higher ranks for the PGCL, right VC, right mRt, and PBC notably change postnatally, while the hub ranks of DR, VTA and PAG remain stable over age within both observed networks. In contrast, the BF, left VC, HY, and Th have higher hub ranks within the whole brain than in the AAN. For this analysis, the one‐month‐old specimen was excluded since the atlas‐based segmentation of cortical regions did not converge at sufficient quality, owing to *postmortem* handling artifacts.

### 3.3 Assessment of Short‐ and Long‐Range Structural Connectivity Development

We computed the number of long‐range and short‐range connections to assess developmental trends in the AAN that is, to identify brainstem regions whose connectivity patterns change with age and to determine whether specific connectivity patterns form age‐dependent spatial clusters. The number of short‐range/long‐range connections was computed based on distance estimates of connections between all 27 seed ROIs of the AAN and the 85 target ROIs. Since structural connectivity estimated by probabilistic tractography may show coarse dependency on distances in large‐scale brain networks (Chang et al. 2022; Morris et al. 2008), short‐range/long‐range estimates are categorized into *highly* probable connections (CP > 0.7), *moderately* probable connections (with 0.7 > CP > 0.5) and *minimally* probable connections (with 0.5 > CP > 0.3) (see Figure 5). Highly probable connections in the DR show more short‐range connections and only display long‐range connections in the moderate probability range. In contrast to the DR, the VTA shows the opposite pattern of more long‐ than short‐range connections. In general, the number of long‐range connections increases with age, becoming visible in infants and adults. In the serotonergic system (light green regions on the y‐axis), there is a higher number of short‐range connections (of moderate and high probability) in the rostral serotonergic system (DR, MnR) in comparison to the caudal system (PGCL, CR). An increase in short‐range connections after the fetal period is also present in the PAG.

### 3.4 AAN Hub Connectogram and Deterministic Tractography Analysis

Core findings of our graph‐based analysis are the identification of the DR as an AAN hub already in the mid‐fetal period, and its role as a hub connecting to cortical regions. In Figure 6 (top panel) the connectograms with DR (as seed) to the 27 AAN ROIs are shown, where CP is encoded in the link thickness. With increasing age, the number of moderate and strong connections from DR to AAN regions is also increasing. The connectivity strength from DR to PGCL, VC, and CR increases postnatally from low to moderate. In the fetal period strong connections are observed from DR to VTA, mRt, and PAG. The 20 and 21wg fetuses begin to show low connection strength from DR to diencephalic regions (Th, BF, HY). Postnatally, strong connections from DR to thalamic regions start emerging and manifesting until adulthood, while strong connections from DR to the hypothalamus are present only in adult cases. The DR does not show strong connections to the BF. The fiber bundles seeded from the DR computed by deterministic tractography are illustrated in Figure 6 (bottom panel) for the 19wg, the 2‐month postnatal, and 60‐year‐old subjects (see Figure S3 in the supplement for data of the remaining individuals). The high density of connections to neighboring regions mirrors our computational estimates of graph‐based measures and short‐length connections. Fewer tracts to parietal and occipital regions are visible in the fetus, but they become more apparent postnatally, with an increasing number of tracts with age. The fetal cases only show frontopontine tracts (FPT) from DR, mainly reaching the forebrain via thalamic regions, as well as parts of the dorsal longitudinal fasciculus (DLF) connecting DR via LC and PAG to the hypothalamus. Also, the superior cerebellar peduncle (SCP) seeded from the DR emerges after birth and shows an increased number of tracts in the adult cases of this study (Figure 6). The observed adult fiber tracts seeded from DR in adults are in concordance with reported FPT, DLF, and SCP data in adults by Zhang et al. (2020). Of note, our demonstration here of FPT and DLF tracts and their involved regions suggests the importance of these connections in the ontogeny of the AAN already in the fetal period.

The connectograms with the ventral tegmental area (VTA) as a seed highlight strong connections to PTg (right R and left L), and DR, and connections of moderate strength to LC, mRt (R + L), PnO, Th (R + L), PBC, MnR in all fetal cases (Figure 7, top panel). In postnatal cases, an increase in connectivity strength from VTA to these regions is observed as is the establishment of moderate connections to CR, VC, PAG, MnR, and PGCL. From postnatal to adult cases, the CP between VTA and HY regions increases from moderate to strong. The fiber bundles seeded from the VTA estimated by deterministic tractography streamlines are illustrated in Figure 7 (bottom panel) for subjects at age 19wg, 2 postnatal months and 60 years (see Figure S4 for data for the remaining individuals). In Figure 7, bilateral fiber bundles connecting the VTA and the rostral and caudal ventral tegmental tracts are already visible in the coronal plane in fetal cases, as well as in the sagittal plane; additionally the tracts contributing to the medial forebrain bundle (MFB) are shown. We observed that the VTA, the region with the highest number of long‐range connections and with a constantly high hub rank across age in the AAN, demonstrated anterior‐frontal streamlines of deterministic tractography first in the fetal cases, while parietal and occipital streamlines appear later in the adult cases.

### 3.5 Sequences of the Ontogeny of the AAN Assessed by Probabilistic Tractography

We employed probabilistic tractography to assess developmental AAN connectivity patterns across the three age groups observed in this study (Figure 8; see Figure S5 for detailed connectivity values per seed region across age groups). We made the observations for the following six defined connectivity types related to the four connectivity groups (*DF, Mi, P, Me*—see Table T1) derived from the previous connectivity matrix computations:

*Core connections* (*yellow*) can be defined as connections that are present in all age groups. We observed that core connections are situated mainly in the pons and midbrain (group *P* and *Mi*). Within the *P* group, the VTA has a high number of core connections, from PTg within the *Mi* group and from *P* connections from LDTg, PBC, DR and MnR. Core connections are established between close neighboring regions within a connectivity group and are not establishing long‐range connections between the caudal and rostral or rostral and diencephalic group. Of note, VTA and DR are already forming hubs in the fetal period in the *P* (DR) and *Mi* (VTA) group.

*Fetal and evolving perinatal connections* are present in the fetal period only (*light blue*), and in the fetal and postnatal group (*pink*). These show symmetric patterns between the left and right brainstem in groups P and Mi connecting LDTg via PnO with MnR as well as LC with PnO.

*Connections in both fetuses and adults* (*purple*) are represented in the left brainstem, and, except for one connection, lie between the VTA and the three seed regions: PBC left, LC left, LDTg left and DR.

*Infant connections* (*red*) evolve unilaterally, with more on the left side, and with increased complexity. Specifically, the left PBC evolves in infants towards an important connection hub in this period with majority of red connections. Strong connectivity develops from the fetal to infant period between the VC and CR and from left PGCL to left PBC and towards the left thalamus, which also receives strong incoming connections from left PTg.

*Postnatal connections* (*green*) emerge after birth and remain present in adults, showing left lateralization and forming a connection hub in the left PnO and mRt. The postnatal connections also preferentially form in the median axis, connecting PAG, VTA, DR, MnR and CR.

*Adult connections* (*blue*) include those present in both adults (solid lines) and consist of more bilaterally symmetric pathways over longer distances. Notably, the right thalamus forms a hub among the adult connections.

*Group‐based observation*: Group Me shows weak interconnections in the fetal period but is strongly connected within Me and to P and Mi from infancy onwards. Long‐range connections from the medulla group to DF, as well as P, Mi to DF, evolve postnatally, while asymmetric left‐lateralized connections in the infant period become bilateral in adults.

## 4 Discussion

Ontogeny is the study of how a living organism develops across the lifespan from conception to adulthood (Tomasello 2019). A basic premise of brain ontogeny is that function emerges in parallel with structure, such that the multi‐faceted aspects of arousal parallel the maturation of the neural networks that mediate it. This inextricable relationship between structure and function is critical to understanding the development of human arousal and the pathogenesis of its disorders.

Here, we address the development of the *structural* neural correlates in 27 key nuclei (nodes) of the brainstem AAN from five perinatal *postmortem* human brains at autopsy using tractography (deterministic and probabilistic) and graph theory, in comparison with two adult brains as indices of maturity.

Because we had a limited age range of brains to study, we could not pinpoint the precise timing of the origin and completeness of awareness‐enabling connections of the AAN as they emerge in toddlers, children, adolescents, and younger adults.

However, from our analysis thus far, it is clear that gestational AAN connectivity does not match the hub connectivity of the adult brain. We emphasize that documenting this emergence is the promise of the technology defined in this study, along with other neuroimaging techniques, including postnatal MRI in vivo.

Our major findings are based primarily on observations and are considered preliminary. Nevertheless, there are demonstrable trends in the developing structural connectivity in this feasibility study that are informative, especially given the lack of such data concerning structural correlates of human arousal and their significance in the literature to date.

Importantly, the key findings of our study are that at mid‐gestation (19‐23wg of a 40wg full‐term human pregnancy), subcortical hubs have formed already—the earliest appearance of human hub formation reported to our knowledge. These hubs already involve key nodes that are known to contribute to the AAN, for example, the DR and MnR (serotonin), and VTA (dopamine), with these neurotransmitters and nuclei promoting arousal. The question arises: How do hubs emerge across the time span of human brain development, and can graph theory help us assess the developmental patterns of this changing network configuration? Specifically, across development do hub properties change (i.e., increase or decrease) in the connectivity strength, number of degrees, betweenness, and/or clustering coefficient, and ultimately, a composited hub rank?

A high VTA hub rank, appearing as early as midgestation in the fetal period and persisting in adulthood, is similar in connectivity probability value to the high hub rank of the DR, which emphasizes the major importance of the dopamine (DA) and 5‐HT systems to human arousal. While the biological bases of the differences in the geometry of hub trajectories remain uncertain, we consider that they may be influenced by the functional efficiency of the specific neurotransmitter receptor and other biophysical dynamics of the network yet to be discovered.

The dopaminergic and serotonergic systems are involved in a wide range of behaviors and physiology, extending from cognition to autonomic functions. Their roles as the so‐called “custodians of neuropsychiatric disease” in modulating behavioral output can be tied back to their overlapping circuits (Niederkofler et al. 2015). Instead of understanding these systems independently, their synergistic roles in arousal are particularly relevant in this study. The observed predominance of both the VTA and DR hubs in human brain ontogeny is striking and goes in line with the findings of Niederkofler et al. (2015) recalling the interaction between the DA and 5‐HT systems in the mouse brain, supported by their considerable anatomical and functional overlap. In their underlying study immunocytochemical visualization indicated that the caudal extension of the VTA intermingles with the rostral region of the DR nucleus and that the majority of 5‐HT neurons in the rostral neuron cluster give rise to ascending axonal projections to the midbrain and forebrain. Additionally, the caudal raphe nuclei send mostly descending axons within the brainstem and toward the spinal cord and there is substantial evidence that both clusters project broadly throughout the rostral and caudal brain (Niederkofler et al. 2015). The presented rat‐ and mouse‐focused study by Niederkofler et al. revealed a similar timeline for the development of dopamine and serotonin axonal pathway projections as suggested for the VTA and DR hubs in our human study. This suggests that the VTA and DR are major and separate subcortical hubs, especially due to their early emergence as hubs in utero. These are potential indications that the VTA and DR play crucial roles in the early and evolutionary function of arousal in the ontogeny of the organism, begging the question of their formation phylogenetically.

The DR contains the highest density of serotonin‐producing neurons and arguably the largest and most complex efferent system of projections in the human brain (Teissier et al. 2017). Studies by Okaty et al. in mice utilizing intersectional genetics, RNA‐sequencing, and subtype‐specific manipulations established that serotonergic neurons in the DR are comprised of highly diverse neuronal subtypes, with varied and heterogeneous axonal connectivity (Okaty et al. 2015). This hub is involved in multiple and disparate functions, such as cognition, affect, aggression, pain, sensory‐motor innervation, sleep state transitions, immune functions, and, of particular relevance to our work here, arousal, especially in response to hypercarbia (Kaur et al. 2020; Linley and Vertes 2019; Ren et al. 2018). Importantly, the DR and MnR produce a complex array of diverse neurotransmitters and neuropeptides, underscoring its integrative roles in brain function (Hornung 2003). Not surprisingly, the DR has been implicated in the pathogenesis of multiple neurological and psychiatric diseases, including addiction, bipolar disorder, major depressive disorder, autism, schizophrenia, and obstructive sleep apnea. Recent experimental evidence further suggests that the MnR hub acts, in the vernacular, as a “central behavioral switchboard” in the brain, uniquely positioned to flexibly control and implement alternative behavioral strategies (e.g., exploring from the environment) (Ahmadlou et al. 2023). In relation to the rest of the AAN seed regions, the location of the DR along the midline of the brainstem may enable more direct short‐range connections to other AAN nodes, a possible consideration for hub formation in the AAN.

Regarding the VTA hub, major functions include affect, award‐seeking behavior, and cognition (Morales and Margolis 2017). Cumulative recent evidence from behavioral, pharmacological, electrophysiological, optogenetic, and chemogenetic experiments demonstrates the VTA's function of arousal as a major role as well (Edlow 2021). VTA lesions in cats are associated with a markedly reduced level of arousal (Jones et al. 1973). Optogenetic stimulation of the VTA promotes recovery from general anesthesia in mice, and can reverse anesthetic‐induced unconsciousness (Taylor et al. 2016). In humans, functional MRI studies have shown that loss of functional connectivity between the VTA and cortical awareness regions highly correlates with decreased levels of consciousness in general anesthesia and traumatic brain injury (Spindler et al. 2021), further elucidating the VTA's central role in the modulation of consciousness. VTA arousal processes are triggered by an increase in dopaminergic tone or by the direct stimulation of dopamine neurons. Pharmacologically, waking can be induced by increasing the availability of synaptic dopamine with consequent saturation of postsynaptic dopamine receptors, for example, by blocking the dopamine transporter to prevent the reuptake of dopamine (Fraigne et al. 2023). The dopaminergic projections from the VTA also play a prominent role in activating REM sleep and, together with the phasic release of dopamine in the basolateral amygdala (BLA), in influencing the timing of REM sleep. How the BLA and VTA dopaminergic neurons communicate to generate REM sleep remains an open question (Fraigne et al. 2023; Hasegawa et al. 2022).

The AAN comprises various neurotransmitter‐specific circuits in the brainstem, hypothalamus, and basal forebrain. While these circuits are thought to be partially redundant, likely to protect survival with fail‐safe assurance during waking and particularly during sleep, each circuit may play different roles in overall arousal to synchronize the entire phenomenon into one homeostatic mechanism (Darnall 2013). Components of arousal in waking or heightened attention are conveyed in specific and even disparate anatomical pathways that mediate different homeostatic responses to hypoxia, hypercarbia, pain, touch, sound, or temperature, to name but a few (Dylag and Martin 2020; Jones 2020; Kinney and Thach 2009; McCulloch et al. 1982; McNamara et al. 1998). There are also likely critical periods in development when distinct maturational changes occur in different arousal‐promoting regions to meet specific clinical needs at particular times. The evidence for this latter concept directly in the human brain, and in human physiology and behavior is admittedly limited (Horne et al. 2004). In our study, however, the concept is supported by the finding of transiently increased connectivity strength to the PBC, an arousal‐promoting region in the AAN, in the early postnatal period.

The PBC in the dorsolateral pons at the junction of the midbrain plays a major role in arousal and the propagation of information from internal and external states to the forebrain to regulate homeostasis and enable a protective response to harmful conditions (Chiang et al. 2019; Pauli et al. 2022). In particular, the PBC receives projections from respiratory chemosensory (e.g., carbon dioxide) pathways from the nucleus of the solitary tract, which in turn project to multiple heterogeneous regions essential to arousal. These regions include the basal forebrain, lateral hypothalamus, midline thalamus, and cerebral cortex (Kaur et al. 2013).

The efferents from the PBC project back to the nucleus of the solitary tract and to the ventrolateral medulla, including the retrotrapezoid nucleus and spinal cord, sites of critical respiratory and autonomic cell groups. Many of these latter brainstem and forebrain areas send efferents back to the PBC. Kaur et al. investigated the glutamatergic signaling of the PBC using animal models and determined that the deletion of the vesicular glutamate transporter 2 gene from glutamatergic neurons in the PBC affects the arousal from sleep in response to CO_2_ and that spontaneous waking is promoted by the medial PBC (Kaur et al. 2013).

In this tractography study, we found that PBC hub connections are only present transiently in the infant and not in the fetal or adult periods. Due to the lack of specimens from late infancy or childhood, we cannot infer the length of this transient time‐frame. The connections of the PBC also evolve asymmetrically with a higher number of strong connections from AAN regions to the PBC on the left during the infant period. PBC left also shows increased strong connections to contralateral AAN ROIs on the right in comparison to the fetal and adult periods. Specifically, the left PBC evolves in infants towards an important connection hub in this immediate postnatal period. The clinical correlates of the transient PBC hub are unknown in this critical developmental period, but we can infer from the PBC's known neural function that it is related to chemosensitivity to carbon dioxide in hypercapnic environments, particularly during prone sleep and with antecedent asphyxia and apnea, as suggested in SIDS (Harper and Kinney 2010). The developmental (neuroplastic) changes in the structural correlates of the changing PBC hub are also unknown but likely due to axonal regression. Indeed, the process of pruning in the first months after birth is responsible for narrowing the number of connections based on activity, strengthening connections of importance, and weakening or disconnecting poor or redundant ones (Kostović et al. 2019).

We are aware of several limitations to our study. It is inherently cross‐sectional and has been established based on a small sample set. Collecting whole brain neurotypical samples in the perinatal period is extremely challenging. However, we have been expanding our network of research collaborators to facilitate access to higher numbers. To increase the number of adolescent and adult samples, we plan to deepen our collaboration with Dr. Edlow's group. Particularly, in the future, we will expand our sample size of all ex vivo brains, extending it across the entire lifespan, thus building upon this preliminary, “baseline” mapping of the maturation of the AAN directly in the human brain.

Due to the nature of the performed analysis, no multiple‐hypothesis correction was performed. The tractography results reported here do not represent “ground‐truth” anatomy of axons; all should be considered inferential. These results are subject to the inherent and well‐known limitations of diffusion MRI tractography (Grisot et al. 2021; Jbabdi and Johansen‐Berg 2011; Jones et al. 2013; Maier‐Hein et al. 2017; Thomas et al. 2014).

It is our overarching goal to advance the understanding of how and why arousal fails in disorders such as, for example, coma, SIDS and seizures. Of particular interest is the emergence of arousal‐promoting circuits contemporaneous with those mediating REM and NREM sleep (beyond the scope of this report). We anticipate that the methods presented here can be streamlined (automated) to become a practical tool in the armamentarium of the neuropathologist in analyzing axonal connectivity at autopsy, employing graph theory mathematics to probe multiple neural network disorders in humans of all ages. This technology has the potential to elucidate network pathology in disorders of arousal in infancy, as postulated for SIDS, the leading cause of postneonatal infant mortality in the United States today (Raven 2018b), for which conventional methods of anatomic neuropathology have been unrevealing. By applying this approach directly to the developing human brain, we believe we have now entered a new frontier in the growing discipline of the brain network‐based autopsy (Edlow et al. 2024).

## Author Contributions

Roxane Licandro performed graph‐based analysis, created corresponding visualization, preprocessing and analysis pipelines. Andre van der Kouwe developed tailored imaging protocols for *postmortem* MRI acquisitions and supported the imaging process, Luiz F. Ferraz da Silva prepared the *postmortem* postnatal specimens for scanning, Camilo Jaimes the fetal brains and Brian L. Edlow the adult brains. All three supported the imaging process. Nathan X. Ngo and William Kelley carried out the image preprocessing and prepared scripts for deterministic and probabilistic tractography together with Lilla Zöllei, Mark Olchanyi and Brian L. Edlow verified the analytical methods and created connectogram visualizations. Mark Olchanyi and Brian L. Edlow annotated all AAN ROIs. Roxane Licandro and Hannah C. Kinney wrote the manuscript with the support from Brian L. Edlow, Lilla Zöllei, Rebecca Folkerth, Robin L. Haynes, Mark Olchanyi. All authors discussed the results and contributed to the final manuscript. Lilla Zöllei supervised and coordinated the project.

## Funding

National Institute of Neurological Disorders and Stroke (U01NS086625 and R21NS109627 (B.L.E)). National Institute of Biomedical Imaging and Bioengineering (1R01EB023281 (B.F.), P41EB015896 (B.R.R.)) National Institute of Child Health and Human Development (5R01HD102616, 5R21HD095338, DP2HD101400 (B.L.E.)) American SIDS Institute. American Academy of Neurology/American Brain Foundation (B.L.E.) James S. McDonnell Foundation (B.L.E.) MIT‐Takeda Fellowship (M.O.) NIH Neuroimaging Training Program (T32) fellowship (5T32EB001680‐19) (M.O.). Computational resources for this research were generously provided by the Massachusetts Life Sciences Center (www.masslifesciences.com/).

## Ethics Statement

When necessary, *postmortem* brains were transferred in accordance with a Material Transfer Agreement (MTA) to the Athinoula A. Martinos Center for Biomedical Imaging, Massachusetts General Hospital (#2021A012114, 2018A015344). This imaging study was approved by the Mass General Brigham Institutional Review Board (2015B000041, 2019P003875, 2010P000243).

## Consent

For brain specimen acquisition, informed consent from surrogate decision‐makers was obtained in compliance with standard operating procedures following respective institutional policies.

## Conflicts of Interest

The authors declare no conflicts of interest.

## Supporting information


**Figure S1:** Image processing workflow for graph‐based analysis of structural connectivity: (1) dMRI pre‐processing and (2) sMRI pre‐processing to annotate ROIs (27 brainstem ROIs).
**Figure S2:** Schematic illustration of how graph‐based measures are computed and used for defining hub regions in a connectivity graph. The degree is computed by the number of edges.
**Figure S3:** Deterministic tractography visualizations of DR seed regions for all subjects.
**Figure S4:** Deterministic tractography visualizations of VTA seed region for all subjects.
**Figure S5:** Sequence visualization of connectivity probability per seed region over age.
**Table T1**. Identified Connectivity Clusters in Structural Connectivity Matrices over Age.

## Data Availability

All data associated with this study are present in the paper or the Supporting Information document. We created a repository on OpenNeuro (https://openneuro.org/datasets/ds005563) to provide all relevant imaging and derived data described in this paper. This link will become active upon the acceptance of our submission. All code used to process the *postmortem* MRI data will be available upon acceptance of this publication via https://github.com/lillazollei/HBM2025.

## Endnotes

^1^

https://fsl.fmrib.ox.ac.uk/fsl/fslwiki/FDT/UserGuide.
