# Supplementary material for: High Resolution Postmortem MRI Discovers Developing Structural Connectivity in the Human Ascending Arousal Network
Source: Hum Brain Mapp. 2025 Nov 29;46(17):e70422. doi: 10.1002/hbm.70422 (PMC12665045; doi:10.1002/hbm.70422)
Supplement: Supplementary file 1 — Figure S1: Image processing workflow for graph‐based analysis of structural connectivity: (1) dMRI pre‐processing and (2) sMRI pre‐processing to annotate ROIs (27 brainstem ROIs). Figure S2: Schematic illustration of how graph‐based measures are computed and used for defining hub regions in a connectivity graph. The degree is computed by the number of edges. Figure S3: Deterministic tractography visualizations of DR seed regions for all subjects. Figure S4: Deterministic tractography visualizations of VTA seed region for all subjects. Figure S5: Sequence visualization of connectivity probability per seed region over age. Table T1. Identified Connectivity Clusters in Structural Connectivity Matrices over Age. [file HBM-46-e70422-s001.pdf]

HIGH RESOLUTION POSTMORTEM MRI DISCOVERS DEVELOPING STRUCTURAL CONNECTIVITY IN THE HUMAN ASCENDING AROUSAL NETWORK

R. Licandro[1,2], M. Olchanyi[1,3], L. F. Ferraz da Silva[4], A. van der Kouwe[1], C. Jaimes[5,7], N. X. Ngo[1], W. Kelley[1], R. Folkerth[6], R. L. Haynes[7], B. L. Edlow[1,3], H.C. Kinney[8], L. Zöllei [1*]

Supplementary

Table T1: *Identified Connectivity Clusters in Structural Connectivity Matrices over Age.*

Connectivity cluster abbreviation and names		Involved Regions
**Me**	Medulla cluster	ParaGigantoCellularis Lateralis - PGCL, Caudal Raphe - CR, Vagal Complex - VC)
**P**	Rostral brainstem – pons cluster	Median Raphe – MnR (serotonergic), Locus coeruleus – LC (noradrenergic), ParaBrachial Complex – PBC (Glutamatergic, Laterodorsal tegmental nucleus – LDTg (cholinergic), Pontis Oralis – PnO (glutamatergic)
**Mi**	Rostral brainstem – midbrain and boarder of pons and midbrain cluster	Pedunculotegmental nucleus – PTg (cholinergic), PeriAqueductal Gray - PAG, mesencephalic Reticulation formation – mRt (glutamatergic), ventral tegmental area – VTA (dopaminergic)
**DF**	Diencephalon/forebrain cluster	Thalamus - Th, Hypothalamus - HY, Basal Forebrain - BF

[1] Laboratories for Computational Neuroimaging - Athinoula A. Martinos Center for Biomedical Imaging – Massachusetts General Hospital and Harvard Medical School, Charlestown, MA, USA.
[2] Computational Imaging Research - Early Life Image Analysis Group, Department of Biomedical Imaging and Image-guided Therapy, Medical University of Vienna, Vienna, AUSTRIA.
[3] Center for Neurotechnology and Neurorecovery, Department of Neurology, Massachusetts General Hospital and Harvard Medical School, Boston, MA, USA
[4] University of São Paulo, Department of Pathology, São Paulo, BRASIL
[5] Department of Radiology, Harvard Medical School, Boston, MA USA
[6] New York University (NYU) Grossman School of Medicine, Department of Forensic Medicine, NY, USA
[7] Department of Pathology, Boston Children's Hospital, Boston, MA, USA
*Corresponding Author

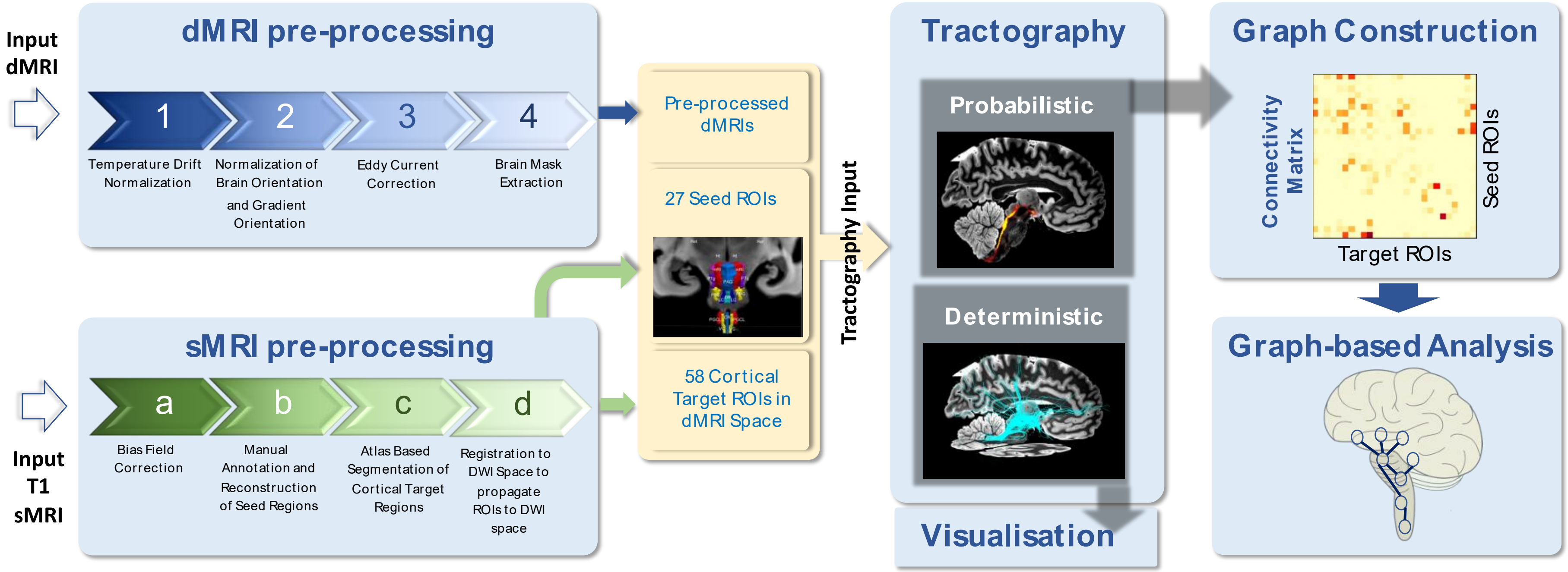

Fig. S1: *Image processing workflow for graph-based analysis of structural connectivity: (1) dMRI pre-processing and (2) sMRI pre-processing to annotate ROIs (27 brainstem ROIs and 58 cortical ROIs) for the subsequent processing steps. Then probabilistic tractography is performed, followed by the construction of a connectivity network, which forms the basis for the graph-based analysis.*

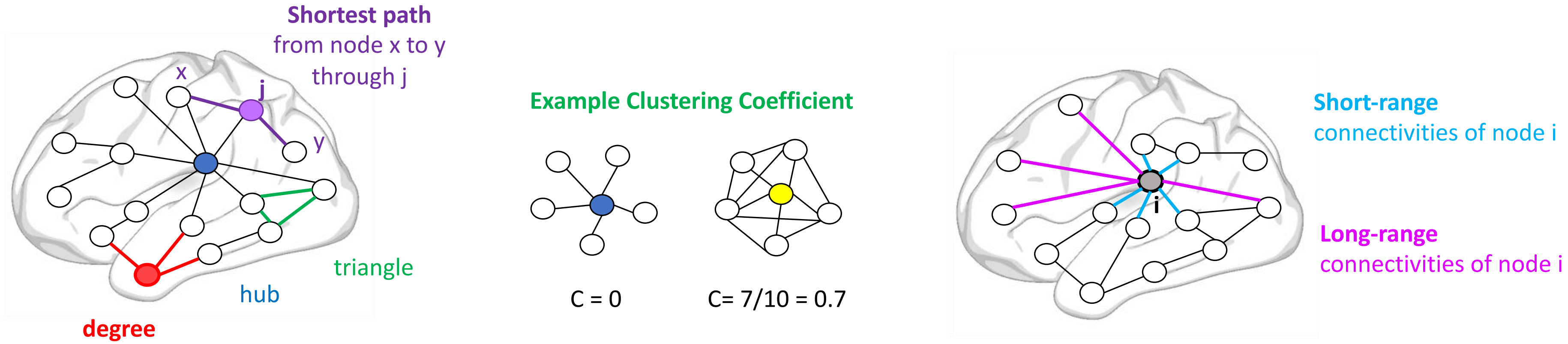

Fig. S2: *Schematic illustration of how graph-based measures are computed and used for defining hub regions in a connectivity graph. The degree is computed by the number of edges connecting to a node. The hub node, visualized in blue on the left brain has degree 8. The edges for computing the degree of the red node (degree=3) are visualized in red. An example of a triangle structure in a graph is visualized in green and analyzed for the computation of the clustering coefficient measure. An example of a shortest path going through the purple node j, is the purple path formed by two purple edges, connecting node x with node y.* ***A hub*** *(see blue node example in the middle)* ***can be seen as a node of high degree, nonbeing part of a cluster*** *(low number of triangles connected to it)* ***and participating in a large number of shortest paths in the network****. On the right an example of short-range connections of node i are visualized in blue (5) and long-range connections (4) in pink.*

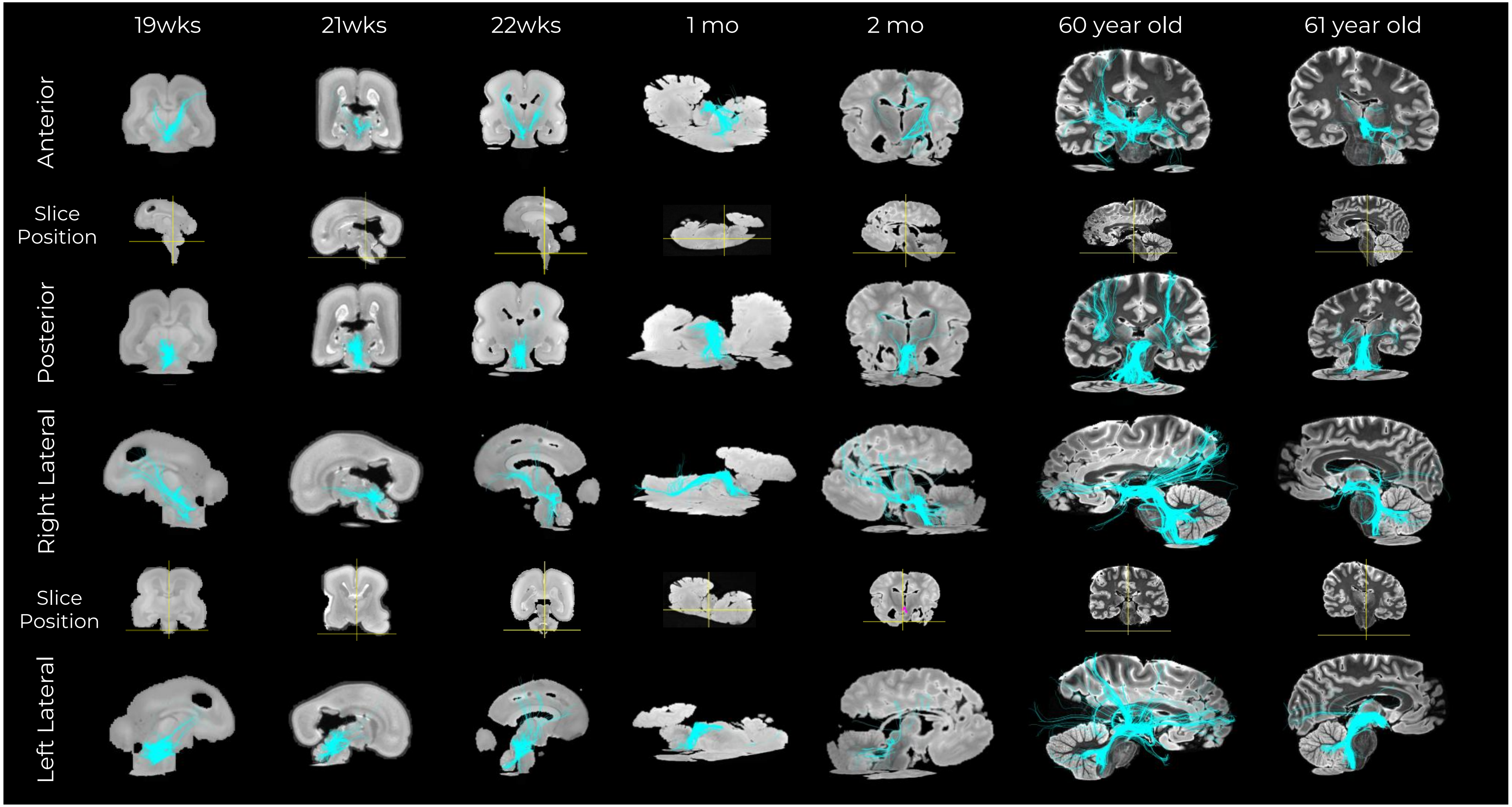

Fig. S3: *Deterministic tractography visualizations of DR seed regions for all subjects*

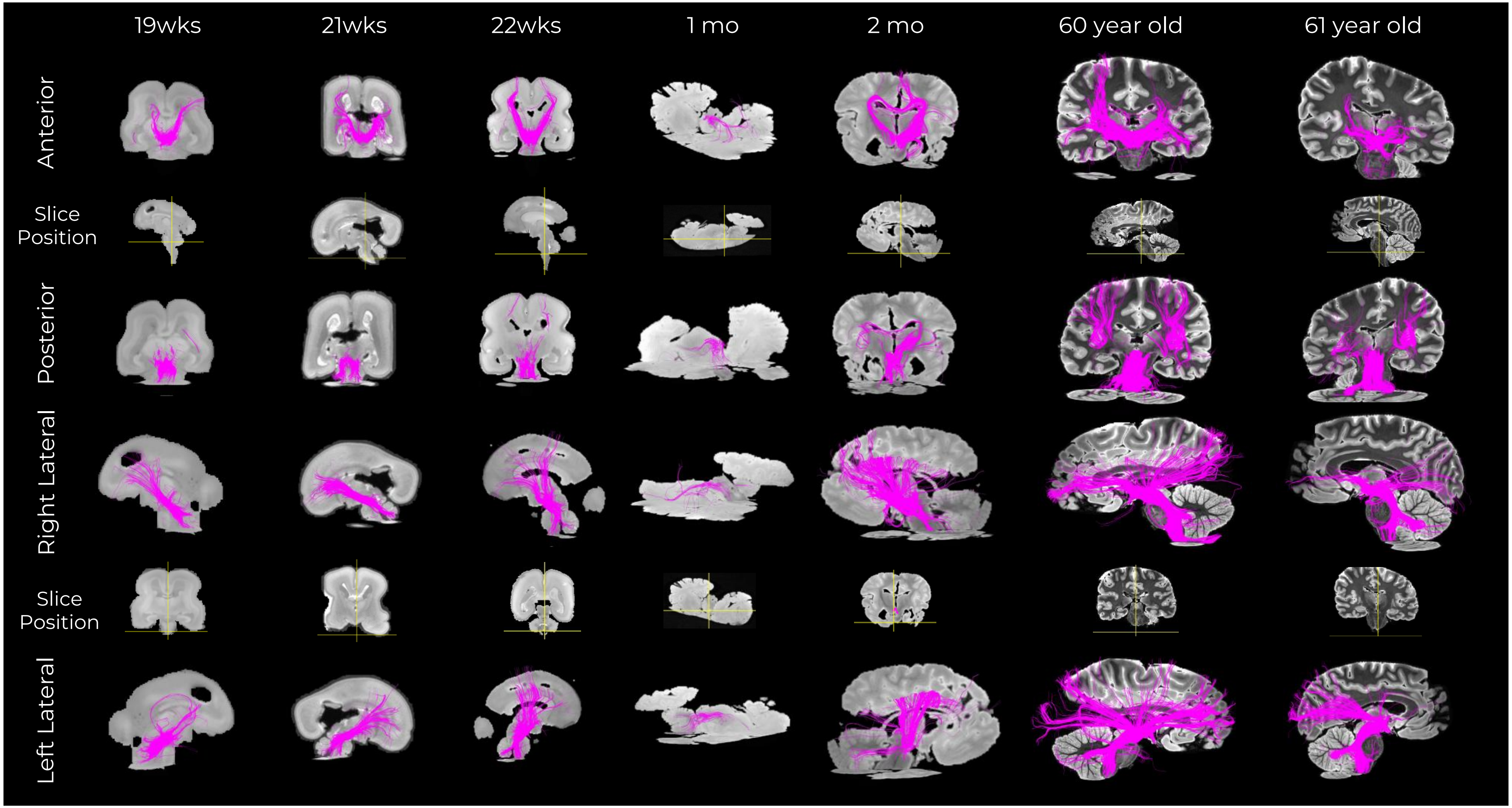

Fig. S4: *Deterministic tractography visualizations of VTA seed region for all subjects*

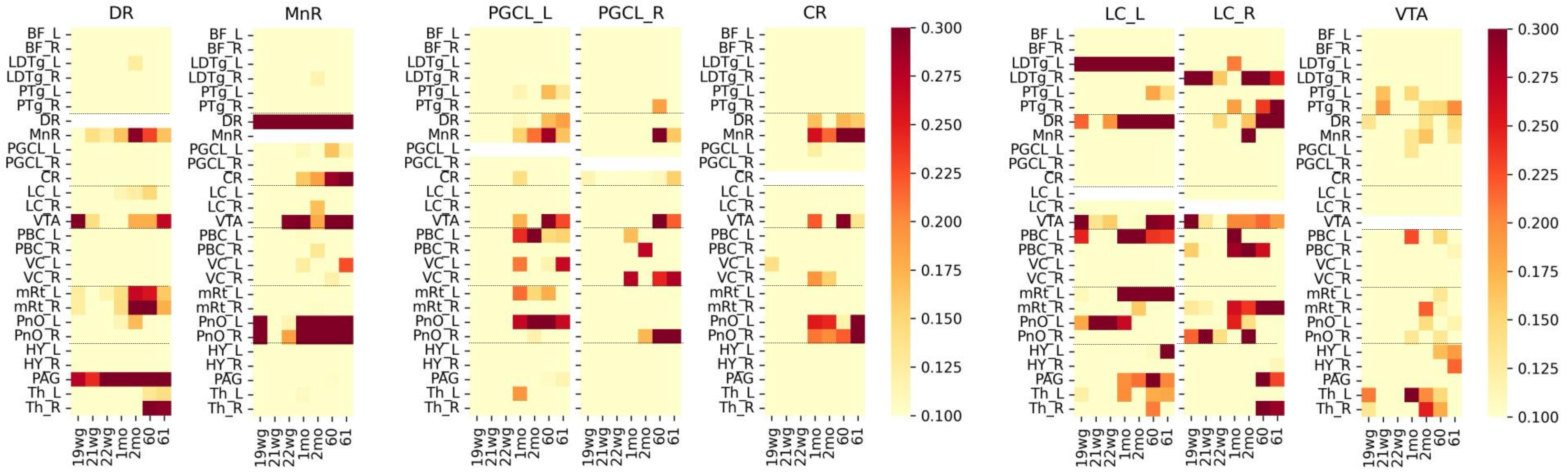

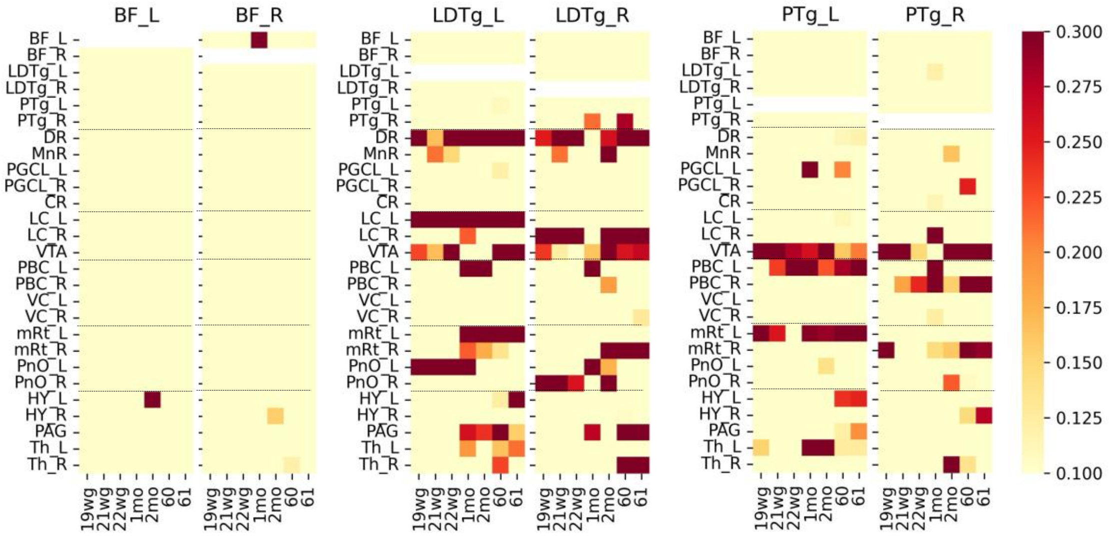

Title of every plot indicates the seed region, y-axis the target regions, x-axis the age, and the value the connectivity probability (dark red… strong connection, yellow… weak connection)

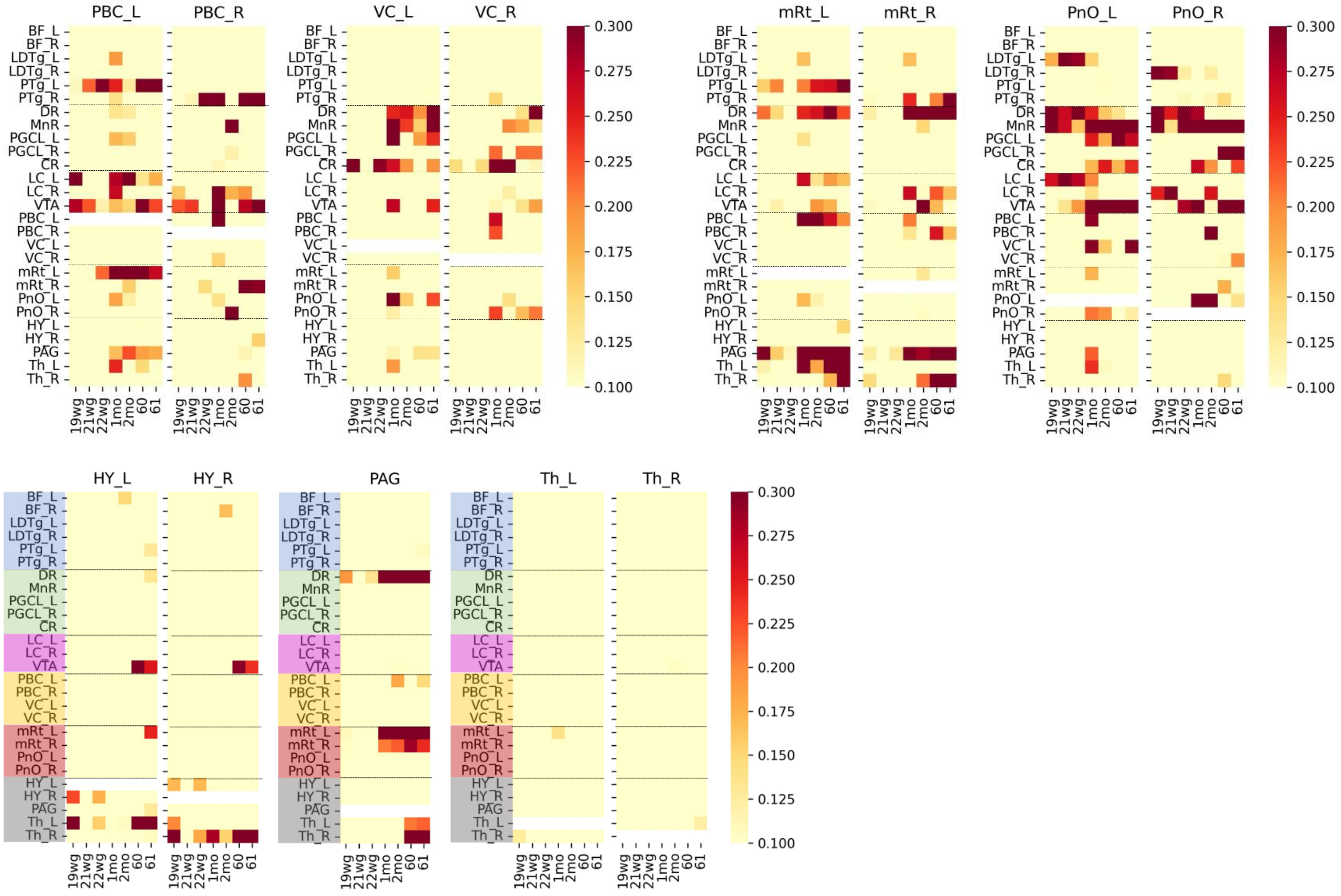

Fig. S5: *Sequence visualization of connectivity probability per seed region over age*
